# Immune‐responsive biodegradable scaffolds for enhancing neutrophil regeneration

**DOI:** 10.1002/btm2.10309

**Published:** 2022-04-19

**Authors:** Matthew D. Kerr, David A. McBride, Wade T. Johnson, Arun K. Chumber, Alexander J. Najibi, Bo Ri Seo, Alexander G. Stafford, David T. Scadden, David J. Mooney, Nisarg J. Shah

**Affiliations:** ^1^ Department of Nanoengineering University of California, San Diego La Jolla California USA; ^2^ Chemical Engineering Program University of California, San Diego La Jolla California USA; ^3^ John A. Paulson School of Engineering and Applied Sciences Harvard University Cambridge Massachusetts USA; ^4^ Wyss Institute for Biologically Inspired Engineering Harvard University Cambridge Massachusetts USA; ^5^ Department of Stem Cell and Regenerative Biology Harvard University Cambridge Massachusetts USA; ^6^ Harvard Stem Cell Institute Cambridge Massachusetts USA; ^7^ Center for Regenerative Medicine Massachusetts General Hospital Boston Massachusetts USA; ^8^ Program in Immunology University of California, San Diego La Jolla California USA

**Keywords:** biomaterials, hematopoietic stem cell transplant, immunodeficiency, neutrophils

## Abstract

Neutrophils are essential effector cells for mediating rapid host defense and their insufficiency arising from therapy‐induced side‐effects, termed neutropenia, can lead to immunodeficiency‐associated complications. In autologous hematopoietic stem cell transplantation (HSCT), neutropenia is a complication that limits therapeutic efficacy. Here, we report the development and in vivo evaluation of an injectable, biodegradable hyaluronic acid (HA)‐based scaffold, termed HA cryogel, with myeloid responsive degradation behavior. In mouse models of immune deficiency, we show that the infiltration of functional myeloid‐lineage cells, specifically neutrophils, is essential to mediate HA cryogel degradation. Post‐HSCT neutropenia in recipient mice delayed degradation of HA cryogels by up to 3 weeks. We harnessed the neutrophil‐responsive degradation to sustain the release of granulocyte colony stimulating factor (G‐CSF) from HA cryogels. Sustained release of G‐CSF from HA cryogels enhanced post‐HSCT neutrophil recovery, comparable to pegylated G‐CSF, which, in turn, accelerated cryogel degradation. HA cryogels are a potential approach for enhancing neutrophils and concurrently assessing immune recovery in neutropenic hosts.

## 1 INTRODUCTION

Neutrophils mediate essential host defense against pathogens and are among the earliest responders in tissue injury.^1^, ^2^, ^3^ Neutrophil deficiency, termed neutropenia, contributes to opportunistic infections and could impair tissue regeneration in affected individuals.^4^, ^5^, ^6^, ^7^ In autologous hematopoietic stem cell transplantation (HSCT) pre‐conditioning myelosuppressive regimens can contribute to a marked transient post‐therapy impairment of neutrophils and render recipients susceptible to immune deficiency‐associated complications for up to several weeks.^6^, ^8^, ^9^, ^10^, ^11^

Post‐HSCT neutrophil regeneration follows successful bone marrow engraftment of transplanted hematopoietic cells,^12^, ^13^ facilitated by granulocyte colony stimulating factor (G‐CSF)‐mediated granulopoiesis of hematopoietic cells.^14^, ^15^, ^16^ Neutropenia is typically treated as an emergency and, in a subset of patients, the risk of neutropenia may be prophylactically addressed with post‐HSCT subcutaneous injection of recombinant human G‐CSF (filgrastim) to facilitate recovery.^6^, ^14^, ^17^, ^18^ Daily injections are used as G‐CSF has a half‐life of a 3–4 h, which can be extended by conjugating G‐CSF with polyethylene glycol (PEGylation).^19^, ^20^ However, immune responses against PEG have been demonstrated to enhance clearance of PEG‐G‐CSF in an antibody‐dependent manner.^21^ As multiple cycles of PEG‐G‐CSF treatment are common, long‐term treatment could be rendered ineffective. Therefore, the development of a sustained release method to deliver G‐CSF while avoiding immune responses against PEG, and concurrently assess neutrophil function could greatly improve the current standard‐of‐care.

Seeking to improve post‐HSCT recovery of neutrophils and simultaneously assess recovery, we developed a biodegradable depot to prophylactically deliver G‐CSF in post‐HSCT recipients. The depot comprised a porous injectable scaffold made by low‐temperature crosslinking, termed cryogelation, of hyaluronic acid (HA), an easily sourced and readily derivatized anionic glycosaminoglycan, termed “HA cryogel.” As a component of the extracellular matrix, endogenous HA is a substrate for degradation by myeloid cells through enzymatic action and by neutrophil‐mediated oxidation.^22^, ^23^, ^24^ Harnessing the immune‐responsiveness of HA, we characterized in vivo degradation of HA cryogels in immune deficient and post‐HSCT mice and identified myeloid cell infiltration in HA cryogels to be key mediators in facilitating degradation, which was significantly reduced or altogether eliminated in mice with severely deficient neutrophil function. Transient but profound post‐HSCT myeloid depletion significantly delayed degradation of HA cryogels until recovery of neutrophils.^25^ As the degradation profile of HA cryogels was responsive to neutrophil recovery, we harnessed encapsulated G‐CSF to facilitate the sustained release, which was mediated by HA cryogel degradation. Neutrophil reconstitution was enhanced in post‐HSCT mice injected with G‐CSF‐encapsulated HA cryogels, comparable to a single dose of PEGylated G‐CSF, which accelerated HA cryogel degradation.

## 2 RESULTS

### 2.1 Synthesis and characterization of HA cryogels

**FIGURE 1:**
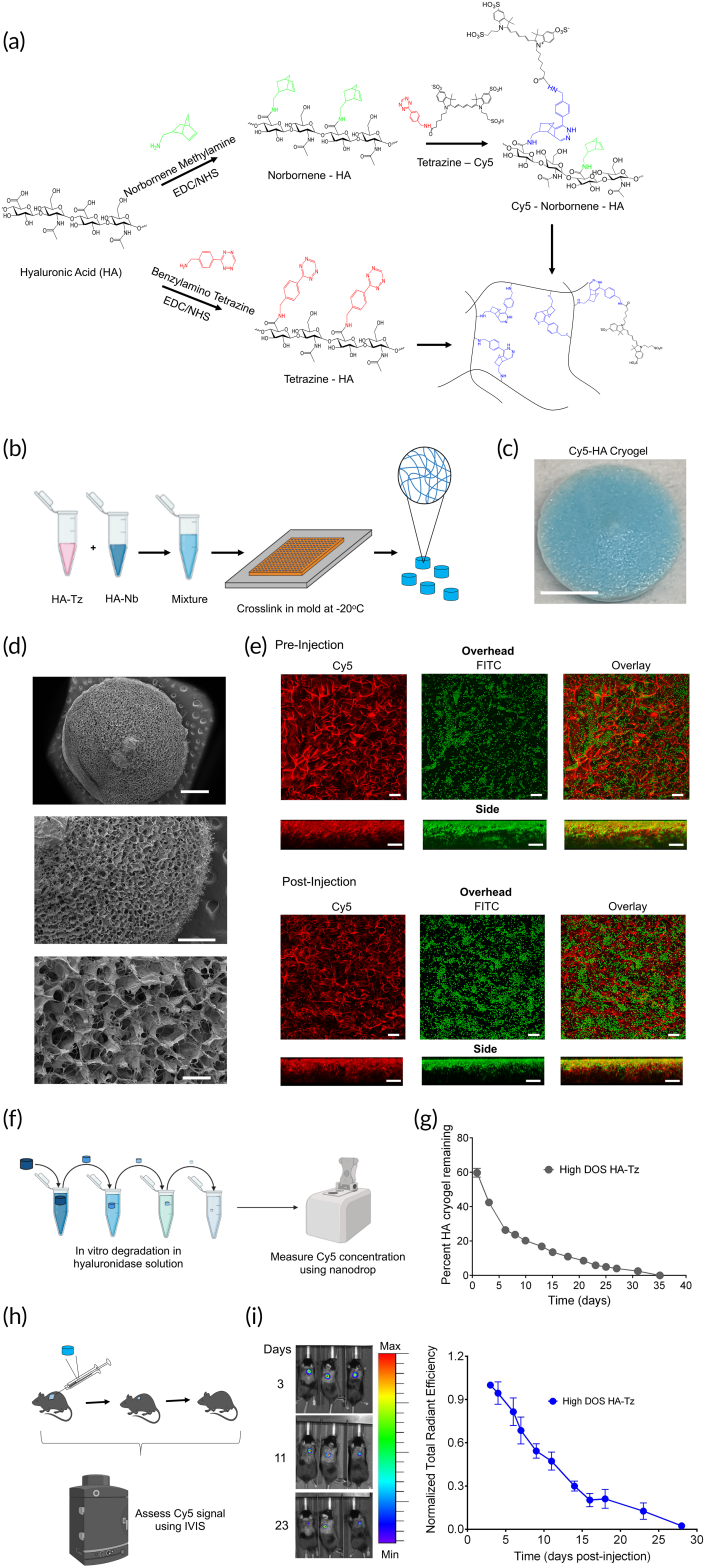
Production and characterization of Cy5‐HA cryogel. (a) Schematic for tetrazine (Tz) and norbornene (Nb) functionalization of HA, Cy5 functionalization of Nb functionalized HA (Cy5‐HA‐Nb) and crosslinking of Tz functionalized HA with Cy5‐HA‐Nb. (b) Schematic for producing Cy5‐HA cryogels. (c) Representative photograph of lyophilized Cy5‐HA cryogel. Scale bar = 1 mm. (d) Representative scanning electron microscopy image depicting Cy5‐HA cryogels. Top scale bar = 1 mm, middle scale bar = 500 μm, bottom scale bar = 100 μm. (e) Confocal microscopy image, overhead and side views, depicting hydrated Cy5‐HA cryogels pre‐ and post‐injection incubated with 10 μm FITC‐labeled microparticles. Scale bar = 100 μm. (f) Schematic depicting workflow for in vitro Cy5‐HA cryogel degradation study. (g) Measuring Cy5‐HA cryogel degradation in vitro by quantifying the Cy5‐signal in supernatant at pre‐determined timepoints normalized to total Cy5‐signal in supernatant across all timepoints. (h) Schematic depicting workflow for in vivo Cy5‐HA cryogel degradation study. (i) Representative in vivo imaging system (IVIS) fluorescence images of gel degradation in mice and measuring Cy5‐HA cryogel degradation in vivo by quantification of total radiant efficiency normalized to initial day 3 timepoint. IVIS Images are on the same scale and analyzed using Living Image Software. Data in (g) represent mean ± s.d. of *n* = 4 HA cryogels. Data in (i) represent mean ± s.e.m. of *n* = 4 HA cryogels. Part of Figure 1b,f,h were created with BioRender.com

Click‐functionalized HA was prepared by conjugating either tetrazine (Tz) amine or norbornene (Nb) methylamine to HA using carbodiimide chemistry. Nb‐ functionalized HA (HA‐Nb) was reacted with Tz‐Cy5 to form Cy5‐labeled HA‐Nb (Cy5‐HA‐Nb) (Figure 1a). Tz amine‐functionalized HA (HA‐Tz) was prepared at 7% degree of substitution (termed high‐DOS). 0.8% DOS HA‐Tz (termed low DOS) was also prepared for comparison. Endotoxin levels of HA‐Tz and Cy5‐HA‐Nb were quantified to be less than 5 endotoxin units/kg, the threshold pyrogenic dose for preclinical species (Table S1).^26^ To maximize polymer concentration while maintaining proper viscosity to achieve mixing, 0.6% w/v aqueous solutions of HA‐Tz and Cy5‐HA‐Nb, pre‐cooled to 4°C, were well mixed in a 1:1 (v/v) ratio by vortexing (Figure 1a). The solution was then pipetted onto individual pre‐cooled (‐20°C) cryomolds (30 μl/mold) and immediately transferred to a −20°C freezer and allowed to freeze (Note S1), to generate Cy5‐HA cryogels (Figure 1b,c).

To characterize Cy5‐HA cryogels, we estimated the swelling ratio by comparing the hydrated versus cast volume and the aqueous mass composition from the wicked mass and fully hydrated mass. The swelling ratio was 1.5 ± 0.1 in both low‐ and high‐DOS Cy5‐HA cryogels (Figure S1a). The aqueous mass composition was 76.3 ± 4.0% and 71.9 ± 2.6% in low‐ and high‐DOS Cy5‐HA cryogels respectively (Figure S1b).

To measure surface porosity of lyophilized Cy5‐HA cryogels, we used scanning electron microscopy (SEM) (Figure 1d; Figure S1c). The surface pore structure images were used to measure the average pore diameter using FIJI, which were between 80–180 μm and 40–90 μm for low‐ and high‐DOS Cy5‐HA cryogels respectively (Figure S1d). To characterize interconnectedness of the Cy5‐HA cryogel pore structure, we incubated fully hydrated low‐ and high‐DOS Cy5‐HA cryogels with Fluorescein isothiocyanate (FITC)‐labeled 10 μm diameter melamine resin particles and imaged using a confocal microscope (Figure 1e; Figure S1e). Since the route of administration of the Cy5‐HA cryogels is through a needle, we repeated this experiment with Cy5‐HA cryogels after injection and observed similar penetration of the FITC‐labeled 10 μm particles (Figure 1e; Figure S1e). Image analysis of z‐stacked images showed co‐localization of the FITC‐labeled 10 μm particles with Cy5‐HA up to a depth of 100 μm below the surface, which was the limit of detection (Figure S1f). Both low‐ and high‐DOS Cy5‐HA cryogels maintained pore morphology and relative surface pore size distribution following lyophilization and rehydration (Figure S1g,h). Cy5‐HA cryogels also maintained shape and structure post‐injection (Movie S1).

To confirm susceptibility of Cy5‐HA cryogels to enzymatic degradation, we used a hyaluronidase‐2 (HYAL2)‐based in vitro assay (Figure 1f). In native HA, HYAL2 cleaves internal beta‐N‐acetyl‐D‐glucosaminidic linkages resulting in fragmentation of HA.^27^ Here, HYAL2 degraded HA cryogels and high DOS Cy5‐HA cryogels degraded at a slower rate compared to the low‐DOS Cy5‐HA cryogels in vitro (Figure 1g; Figure S2a). To assess in vivo degradation, low‐ and high‐DOS Cy5‐HA cryogels were injected subcutaneously in the hind flank of C57Bl/6J (B6) mice and degradation was measured using in vivo imaging system (IVIS) fluorescence spectroscopy (Figure 1h). In contrast to in vitro degradation, both low‐ and high‐DOS Cy5‐HA cryogels degraded at a similar rate (Figure 1i; Figure S2b). This observation, together with the finding of a similar pore size distribution in hydrated low‐ and high‐DOS HA cryogels (Figure S1h), supported the selection of one of the types of HA cryogels for subsequent experiments, and we selected high‐DOS HA cryogels. To characterize if HA cryogels made from different batches of derivatized HA affected in vivo degradation, we compared degradation of Cy5‐HA cryogels made from three distinct batches of Cy5‐HA‐Nb and HA‐Tz and confirmed that all Cy5‐HA‐cryogels degraded at a similar rate (Figure S2c).

### 2.2 Depletion of immune cell subsets affects cellular infiltration into HA cryogels

**FIGURE 2:**
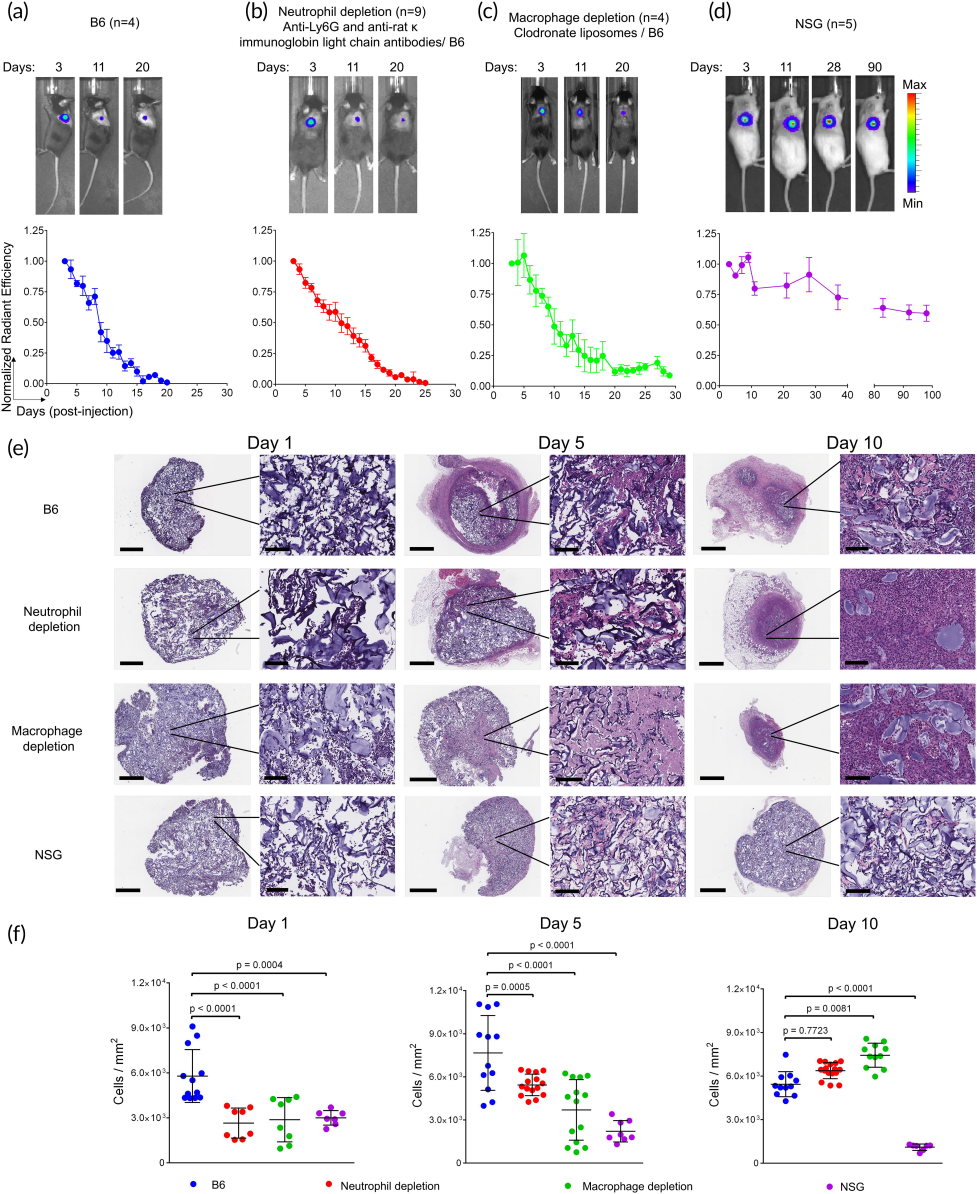
Cy5‐HA cryogel degradation in immunodeficient mice. Representative in vivo imaging system (IVIS) fluorescence images of Cy5‐HA cryogel degradation and quantification by measuring total radiant efficiency normalized to initial day 3 timepoint of (a) untreated B6 mice, (b) neutrophil depleted B6 mice, (c) macrophage depleted B6 mice, and (d) NSG mice. IVIS Images are on the same scale and analyzed using Living Image Software. (e) Hematoxylin and eosin (H&E) stained histological sections of explanted Cy5‐HA cryogels from the above groups, at days 1, 5, and 10 post‐injection. Full view scale bar = 800 μm, magnified scale bar = 100 μm. (f) Quantification of cellular density in the sections from (e). Data in (a–d) represent mean ± s.e.m. of *n* = 4–9 and are representative of at least two separate experiments. Data in (f) represent mean ± s.d. of *n* = 7–12 and were compared using student's *t*‐test

**FIGURE 3:**
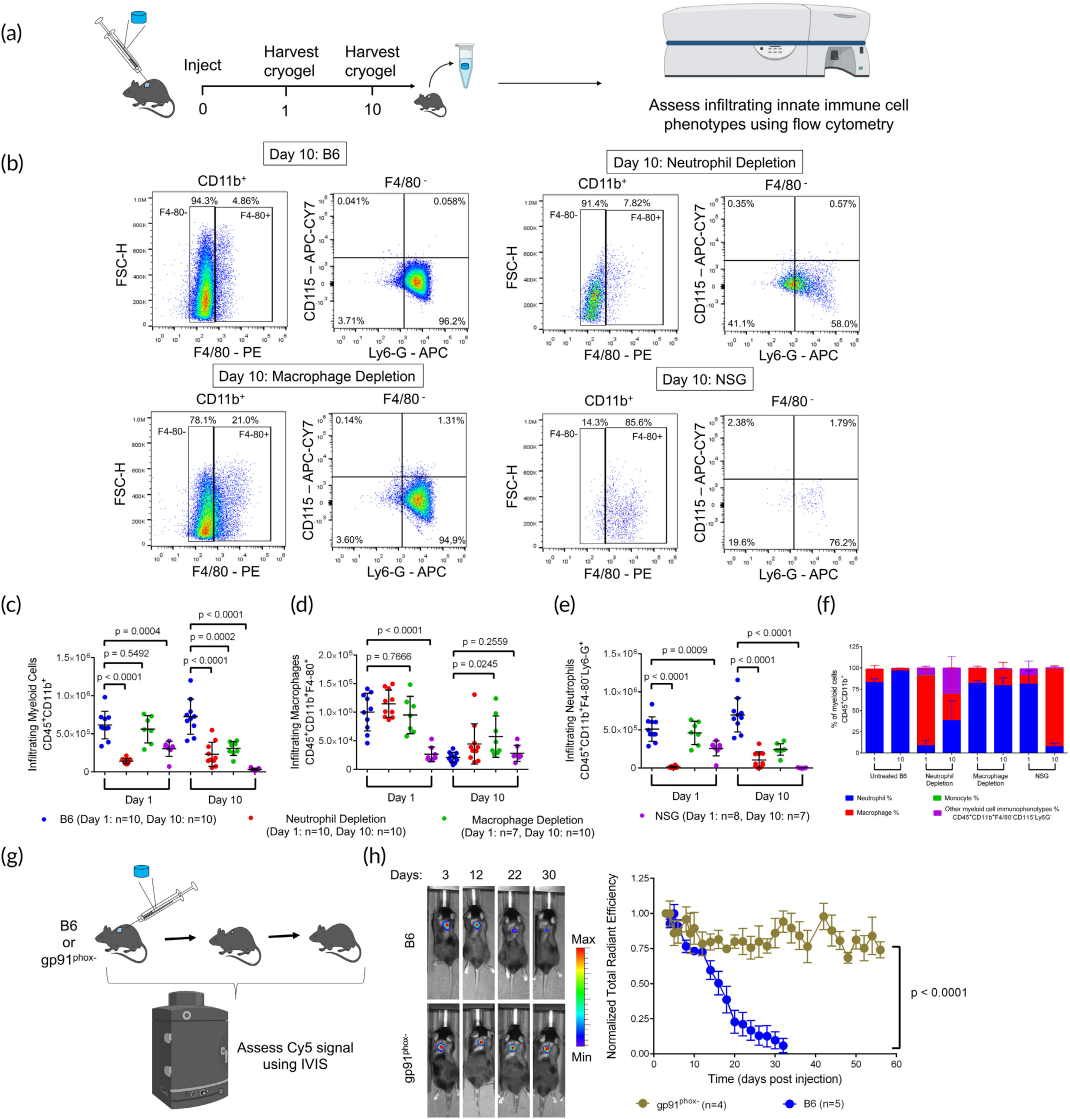
Assessment of innate immune cell infiltration into Cy5‐HA cryogels. (a) Schematic for the quantification of innate immune cell content in Cy5‐HA cryogels. (b) Representative flow cytometry plots depicting gating strategy to determine cellular identity of CD45^+^ CD11b^+^ F4/80^+^ (macrophage) cells, CD45^+^ CD11b^+^ F4/80^−^ Ly6G^+^ (neutrophil) cells, and CD45^+^ CD11b^+^ F4/80^−^ Ly6G^−^ CD115^+^ (monocyte) cells in untreated B6 mice, anti‐Ly6G and anti‐rat κ immunoglobulin light chain antibody treated B6 mice, clodronate liposome treated B6 mice, and NSG mice. (c–e) Quantification of total number of (c) CD45^+^ CD11b^+^ (myeloid) cells, (d) macrophages, and (e) neutrophils infiltrating HA cryogels in untreated B6 mice, anti‐Ly6G and anti‐rat κ immunoglobulin light chain antibody treated B6 mice, clodronate liposome treated B6 mice, and NSG mice. (f) Infiltrating immune cell lineages plotted as a percentage of myeloid cells in untreated, neutrophil depleted, macrophage depleted, and NSG mice. (g) Schematic depicting workflow for in vivo Cy5‐HA cryogel degradation study with gp91^phox−^ mice. (h) Representative in vivo imaging system (IVIS) fluorescence images of gel degradation and quantification by measuring total radiant efficiency normalized to initial day 3 timepoint of gp91^phox−^ mice and B6 mice. IVIS Images are on the same scale and analyzed using Living Image Software. Data in (c–f) represent mean ± s.d. of *n* = 7–10 Cy5‐HA cryogels, are representative of at least two separate experiments. Data in (c–e) were compared using student's *t*‐test. Data in (h) represent mean ± s.e.m. of *n* = 4–5 and were compared using two‐way ANOVA with Bonferroni's multiple comparison test. Parts of Figure 3a,h were created with BioRender.com

As the HSCT pre‐conditioning regimen depletes all immune cell lineages, we first sought to measure the effect of immune depletion on HA cryogel degradation. Cy5‐HA cryogels were subcutaneously injected into the hind flank of untreated B6 mice (Figure 2a) and the degradation profile was compared to that in B6 mice receiving (i) anti‐Ly6G and anti‐rat κ immunoglobulin light chain antibodies to deplete neutrophils (~98% efficiency) (Figure 2b), (ii) clodronate liposomes to deplete monocytes/macrophages (80%–95% efficiency) (Figure 2c), (iii) anti‐CD4 and anti‐CD8 antibodies to deplete T cells (~99% efficiency) (Figure S3a), (iv) anti‐B220 to transiently deplete B‐cells (99% efficiency up to 4 days) (Figure S3b) and immune deficient NOD.Cg‐Prkdcscid Il2rgtm1Wjl/SzJ (NSG) mice (Figure 2d). The durability of depletion was assessed by measuring peripheral blood cellularity throughout the duration of the degradation study (Figure S3c–l; Table S2). In untreated immune competent mice, the average half‐life of Cy5‐HA cryogels, quantified as the time to achieve a 50% reduction in fluorescence intensity, was about 9.5 days (Figure 3m). The average half‐life in the macrophage, neutrophil, T cell, and B cell depleted mice was similar at about 11.8, 11.3, 9.6, and 10.2 days, respectively (Figure 3m). In contrast, only a 35% reduction in Cy5 signal intensity was measured after 3 months in the NSG mice (Figure 2d). Retrieval of Cy5‐HA cryogels from sacrificed mice at the endpoint confirmed that the gels had minimally degraded (Figure S3n).

To assess cellular infiltration and the foreign body response, Cy5‐HA cryogels were explanted from the above groups at 1‐, 5‐, and 10‐days post‐injection and stained using haemotoxylin and eosin (H&E). In Cy5‐HA cryogels retrieved from all groups except NSG mice, the total cellularity increased from day 1 to 10 and formed a distinct capsule encapsulating the HA cryogel, indicative of a foreign body response (Figure 2e; Figure S4a). In NSG mice, some infiltrates were quantified on day 1, however there was no appreciable increase in cellularity at the later timepoints or a capsule by day 10 (Figure 2e). H&E slides were further analyzed to quantify the cell density in the different groups. Differences in infiltrates between the untreated and all immunodepleted B6 mice were significant at the earlier timepoints, and either increased or remained constant in all immunodepleted B6 (Figure 2f; Figure S4b). In contrast, cell infiltrates in HA cryogels retrieved from NSG mice reduced steadily and were 80% lower than untreated B6 mice by day 10 (Figure 2f).

To identify the immune cells contributing to HA cryogel degradation, we quantified cell infiltrates in the Cy5‐HA cryogels 1‐ and 10‐ days post‐injection using flow cytometry in untreated and immune depleted B6 and NSG mice (Figure 3a,b; Figure S5a,b). Viability of infiltrating cells, quantified by negative AnnexinV staining, was consistently greater than 95% in all groups (Figure S5c).

Infiltration of total CD45^+^CD11b^+^ (myeloid) cells into HA cryogels of untreated B6 mice and T cell depleted B6 mice were similar after 1‐ and 10‐days post‐injection (Figure S5d). While there were comparable myeloid cells in HA cryogels retrieved 1‐day post‐injection from B cell depleted B6 mice, by day 10 the number was about 66% lower than in untreated B6 mice (Figure S5e). Similarly, myeloid cell infiltration in Cy5‐HA cryogels retrieved 1‐day post‐injection from macrophage depleted mice was unaffected, however by day 10, the number of infiltrating myeloid cells was about 58% lower than untreated B6 mice (Figure 3c). Neutrophil depletion in B6 mice reduced the total number of myeloid cells in Cy5‐HA cryogels compared with the untreated B6 mice by about 77% 1‐day and 68% 10‐days post‐injection respectively (Figure 3c). In NSG mice myeloid cell infiltration in HA cryogels was 51% lower 1‐day and 91% lower 10‐days post‐injection as compared to untreated B6 mice (Figure 3c).

CD45^+^CD11b^+^F4/80^+^ (macrophage) infiltration in Cy5‐HA cryogels retrieved from all groups except NSG mice reduced from 1‐ to 10‐days (Figure 3d; Figure S5e). Intraperitoneal (i.p.) administration of clodronate liposomes minimally affected macrophage infiltration in Cy5‐HA cryogels even though it was effective in depleting peripheral blood monocytes (Figure 3d; Figure S3g,h). In NSG mice, macrophage infiltration was 74% lower than in the untreated B6 mice on day 1 and remained unchanged 10‐days post‐injection (Figure 3d).

In CD45^+^CD11b^+^F4/80^−^Ly6G^+^ (neutrophil) depleted B6 mice, an additional intracellular Ly6G staining step was included, as the method of neutrophil depletion is known to induce internalization of the Ly6G receptor (Figure S5f).^28^ Neutrophil infiltration in Cy5‐HA cryogels retrieved from untreated B6 and T cell depleted B6 mice was comparable between 1‐ and 10‐days post‐injection (Figure 3e; Figure S5g). B cell depletion did not affect the initial neutrophil infiltration 1‐day post‐injection compared with untreated controls but reduced the number of infiltrating neutrophils by 10‐days post‐injection (Figure S5g). As expected, neutrophil depletion significantly reduced initial neutrophil infiltration, by about 97%, compared to the untreated control. In this group, neutrophils constituted less than 50% of infiltrating myeloid cells at all timepoints assessed (Figure 3f). Despite an increase by day 10, attributable to the internalization of the Ly6G receptor which led to an approximate fourfold increase in the infiltrating neutrophil fraction (Figure S5f), the number of infiltrating neutrophils were still 84% lower compared with untreated B6 mice (Figure 3e). Macrophage depletion did not affect the initial neutrophil infiltration 1‐day post‐injection compared with untreated B6 mice but reduced the number of infiltrating neutrophils by 65% compared with untreated B6 mice by day 10. HA cryogels retrieved from NSG mice had 50% fewer neutrophils than those from untreated B6 mice on day 1 and very few to none were found by day 10 post‐injection (Figure 3e). In NSG mice neutrophils constituted over 90% of total myeloid cells on day 1 but decreased to about 8% by day 10 (Figure 3f). This observation along with minimal Cy5‐HA cryogel degradation in NSG mice (Figure 2d), supported a key role of functional neutrophils in mediating degradation. In all groups, the infiltration of CD45^+^CD11b^+^F4/80^−^Ly6G^−^CD115^+^ (monocyte) cells were minimal and constituted a negligible portion of total infiltrating myeloid cells (Figure S5h,i).

To provide additional confirmation of infiltrating neutrophils and macrophages, we used immunohistochemical (IHC) staining to assess for Ly6G^+^ and F4/80^+^ cells respectively in untreated B6 mice and NSG mice at 1‐, 5‐, and 10‐days post‐injection. Staining on day 1 corroborated the flow cytometry data in that there were more neutrophils than macrophages within the Cy5‐HA cryogels (Figure 5i; Figure S6a). On subsequent days, non‐specific debris precluded accurate assessment in Cy5‐HA cryogels retrieved from B6 mice (Figure S6a). As a result of non‐specific staining of debris at later timepoints, IHC was only conducted on Cy5‐HA cryogels excised 1‐day after injection in macrophage depleted, neutrophil depleted, T cell depleted, and B cell depleted mice. Staining of these samples confirmed the presence of both Ly6G and F4/80 in Cy5‐HA cryogels confirming flow cytometry data (Figures S5i and S6b). In NSG mice, IHC staining of Ly6G^+^ and F4/80^+^ cells followed the results from flow cytometry analysis. Significantly more neutrophils than macrophages in Cy5‐HA cryogels were observed 1‐day after injection (Figure S6c). On day 5, there were significant macrophage and neutrophil infiltrates (Figure S6c) and by day 10, the neutrophil infiltration reduced significantly as expected from flow cytometry analysis (Figures S5h and S6c).

To further characterize the role of functional neutrophils, we compared the degradation of Cy5‐HA cryogels in B6 and B6.129S‐Cybb^tm1Din^ (gp91^phox−^) mice. Affected hemizygous male gp91^phox−^ mice have a defect in the NADPH oxidase enzyme, which renders mice deficient in neutrophil function through the production of reactive oxygen species.^29^, ^30^ Cy5‐HA cryogels were injected in gp91^phox−^ mice and B6 mice and degradation was quantified using IVIS (Figure 3g). Cy5‐HA cryogels did not degrade appreciably in the gp91^phox−^ over the course of the 2‐month study whereas the Cy5‐HA cryogels in B6 mice degraded within 4 weeks, as expected (Figure 3h).

Taken together, these results suggest that inducing immune deficiency by depletion affects cell infiltration in Cy5‐HA cryogels but does not affect degradation. However, deficiencies which functionally impair neutrophils, modeled by NSG and gp91^phox−^ mice are sufficient to significantly affect Cy5‐HA cryogel degradation.

### 2.3 HA cryogels are neutrophil responsive in post‐HSCT mice

**FIGURE 4:**
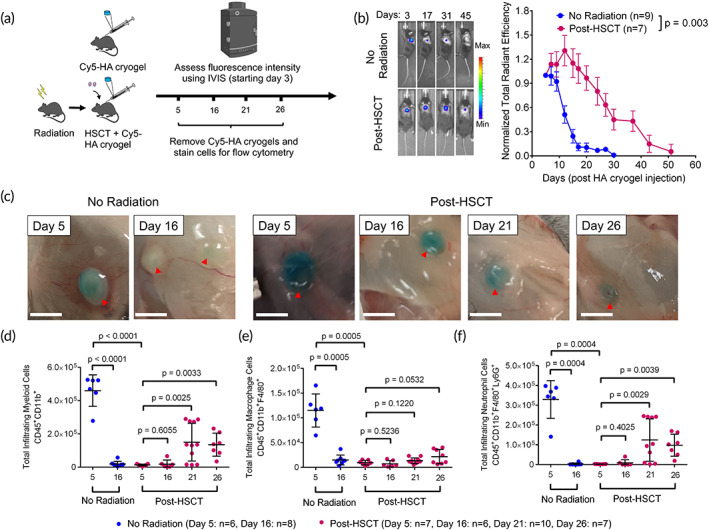
Degradation kinetics of HA cryogels is impaired during transient immunodeficiency following hematopoietic stem cell transplantation (HSCT). (a) Schematic depicting workflow for quantification of Cy5‐HA cryogel degradation and innate immune cell infiltration in control (nonirradiated mice that do not receive a transplant) and post‐HSCT B6 mice. (b) Representative in vivo imaging system fluorescence images of gel degradation in nonirradiated and post‐HSCT mice. Tracking gel degradation by quantification of total radiant efficiency normalized to initial day 3 timepoint. (c) Photograph of Cy5‐HA cryogels in nonirradiated mice 5‐ and 16‐days post‐injection and post‐HSCT mice on days 5, 16, 21, and 26. (d–f) Cell infiltration of (d) CD45^+^CD11b^+^ (myeloid) cells, (e) CD45^+^CD11b^+^ F4/80^+^ (macrophage) cells, and (f) CD45^+^ CD11b^+^ F4/80^−^ Ly6G^+^ (neutrophil) cells into HA cryogels in nonirradiated mice 5‐ and 16‐days post‐injection and 5‐, 16‐, 21‐, and 26‐days post‐HSCT. Data in (b) represent mean ± s.e.m. of *n* = 7–9 mice and is representative of at least two separate experiments. Data in (d–f) represent mean ± s.d. of *n* = 6–10 HA cryogels and are representative of at least two separate experiments. Data in (b) were compared using two‐way ANOVA with Bonferroni's multiple comparison test. Data in (d–f) were compared using student's *t*‐test. Part of Figure 4a was created with BioRender.com

We next quantified Cy5‐HA cryogel degradation in post‐HSCT mice. B6 recipients were irradiated 48 h prior to i.v. injection of lineage depleted hematopoietic stem cells (2 × 10^5^ cells, ~87% depleted) isolated from bone marrow of syngeneic B6 donor mice (Figure S7a). Concurrently, B6 recipients and control mice (B6, non‐irradiated that do not receive a transplant) were injected subcutaneously with Cy5‐HA cryogels, and the degradation rate was compared (Figure 4a). In contrast to nonirradiated mice, a steady fluorescence signal was quantified for about 20 days in post‐HSCT mice after which it decreased, corresponding to HA cryogel degradation, at a rate comparable to that in nonirradiated mice. The time interval to 50% of the initial fluorescence intensity was approximately 30 days in post‐HSCT mice whereas in nonirradiated mice, a comparable decrease was achieved by day 13 (Figure 4b; Figure S7b).

To quantify infiltrating myeloid subsets, Cy5‐HA cryogels were excised on days 5 and 16 post‐injection in nonirradiated mice and excised on days 5, 16, 21, and 26 in post‐HSCT mice (Figure 4c). Viability of infiltrating cells, quantified by negative AnnexinV staining, was initially lower 5‐ and 16‐days post‐injection, and increased by day 21 (Figure S7c). In nonirradiated B6 mice, the number of infiltrating myeloid cells decreased by 96% from days 5 to 16 post‐injection (Figure 4d), mirroring near‐complete Cy5‐HA cryogel degradation (Figure 4b; Figure S7b). In contrast, myeloid cell infiltration in Cy5‐HA cryogels was significantly delayed and 97% lower than that of the nonirradiated group, 5 days post‐injection. In post‐HSCT mice, appreciable myeloid infiltration was not quantified until about day 21 post‐HSCT, which was still 67% lower when compared with HA cryogels from nonirradiated mice on day 5 (Figure 4d).

Macrophage infiltration in Cy5‐HA cryogels in nonirradiated mice decreased 87% from days 5 to 16 (Figure 4e). On day 5 in post‐HSCT mice, macrophage infiltration in Cy5‐HA cryogels was reduced by about 92% compared to nonirradiated mice. By day 26, infiltrating macrophages in some post‐HSCT mice were quantified but remained significantly lower than macrophage infiltration on day 5 in nonirradiated mice (Figure 4e).

Neutrophils constituted a majority of the myeloid cells in Cy5‐HA cryogels in nonirradiated mice on day 5, but not by day 16 (Figure 4f) when the majority of myeloid cells were macrophages (Figure S7d). In contrast, very few cells were in HA cryogels retrieved on day 5 in post‐HSCT mice with a near absence of neutrophils, in contrast with nonirradiated mice at the same timepoint. Neutrophil infiltration in Cy5‐HA cryogels was quantified 21 days post‐injection but was still 62% lower than on day 5 in nonirradiated mice (Figure 4f). In post‐HSCT mice, macrophages constituted most of the cell infiltrates 5‐ and 16‐days after injection, whereas a majority of myeloid cells were neutrophils on days 21 and 26 (Figure S7d). This data supports that irradiation reduces myeloid infiltration in Cy5‐HA cryogels, delays cryogel degradation, and degradation coincides with neutrophil recovery (Figure S7e).

To assess whether the uniqueness of the results could be attributed to the HA cryogels, we compared the results with hydrolytically degradable oxidized alginate (OxAlg), also functionalized with Tz and Nb (Figure S7f). Unlike HA, OxAlg is not a substrate for endogenous enzymes.^31^, ^32^ Tz‐functionalized OxAlg was functionalized with Cy5 and Cy5‐OxAlg cryogels were formed in the same manner as high‐DOS Cy5‐HA cryogels. In vitro, Cy5‐OxAlg cryogels fully degraded in 1× PBS over 9‐days (Figure S7g). In contrast to Cy5‐HA cryogels, Cy5‐OxAlg cryogels injected in B6 and post‐HSCT B6 mice degraded rapidly at a comparable rate, with approximately 70% reduction in fluorescence signal within 24 h post‐injection (Figure S7h).

### 2.4 HA cryogels sustain G‐CSF delivery and enhance post‐HSCT reconstitution of neutrophils

**FIGURE 5:**
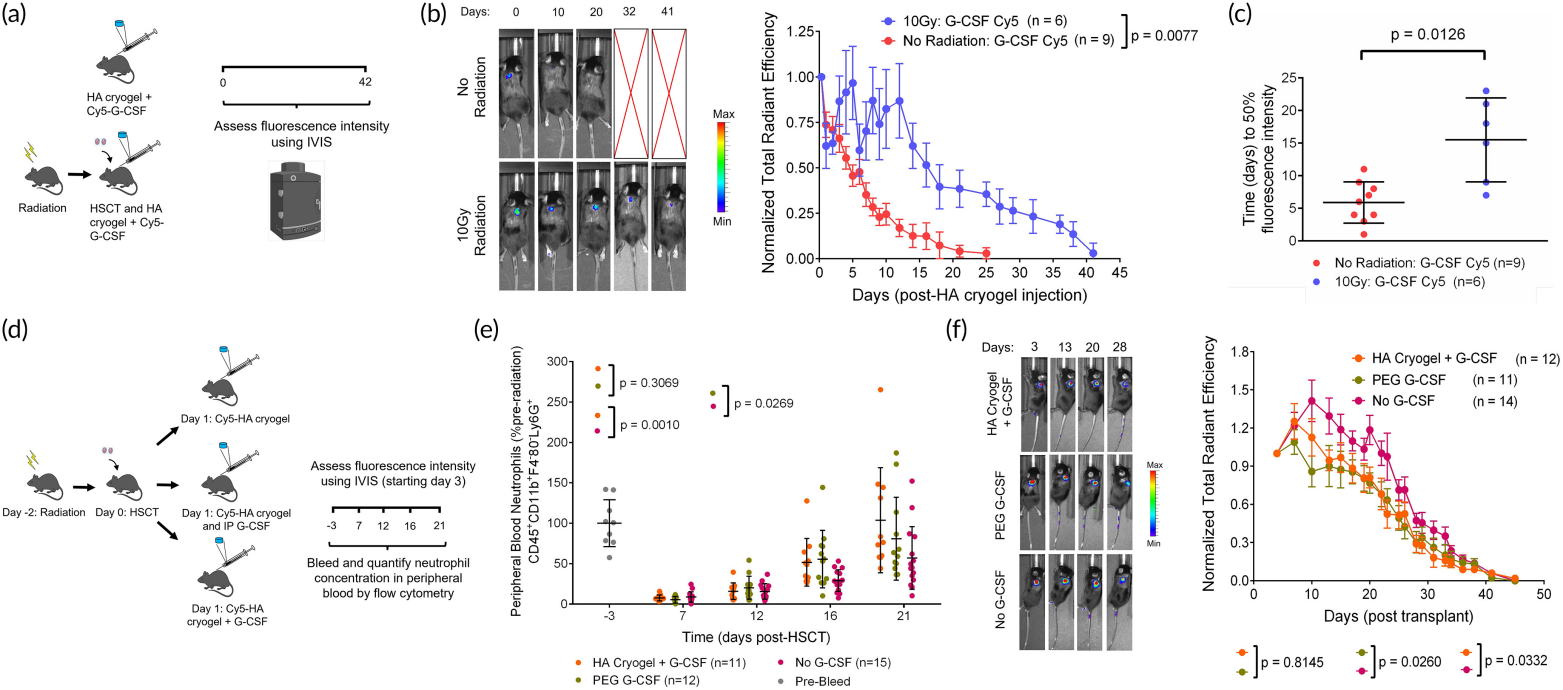
Enhanced reconstitution of peripheral blood neutrophil cells. (a) Schematic depicting outline of study to quantify Cy5 G‐CSF release from HA cryogels in nonirradiated, non‐transplanted B6 mice and post‐hematopoietic stem cell transplantation (HSCT) B6 mice. (b) Representative in vivo imaging system (IVIS) fluorescence images of Cy5 G‐CSF release from HA cryogels and quantification by measuring total radiant efficiency normalized to initial 8‐h timepoint. IVIS Images are on the same scale and analyzed using Living Image Software. (c) Time to 50% fluorescence intensity for Cy5 G‐CSF encapsulated within HA cryogels in nonirradiated and post‐HSCT mice. (d) Schematic depicting outline of study to quantify neutrophil reconstitution rate and Cy5‐HA cryogel degradation rate in post‐HSCT mice using G‐CSF encapsulated Cy5‐HA cryogels. (e) Peripheral blood reconstitution of neutrophils in post‐HSCT mice, normalized to pre‐irradiation neutrophil counts from a random subset of mice. (f) Representative IVIS fluorescence images of gel degradation in mice and measuring Cy5‐HA cryogel degradation in vivo by quantification of total radiant efficiency normalized to initial day 3 timepoint. IVIS Images are on the same scale and analyzed using Living Image Software. Data in (b) represent mean ± s.e.m. of *n* = 6–9 mice. Data in (c) represent mean ± s.d. of *n* = 6–9 mice. Data in (e) represent mean ± s.d. of *n* = 11–15 mice and is representative of at least two separate experiments. Data in (f) represent ± s.e.m. of *n* = 11–14 Cy5‐HA cryogels and is representative of at least two separate experiments. Data in (b and f) were compared using two‐way ANOVA with Bonferroni's multiple comparison test. Data in (c) were compared using student's *t*‐test. Data in (e) were compared using mixed‐effect regression model with random intercepts. Parts of Figure 5a,d were created with BioRender.com

We sought to leverage the delay in post‐HSCT degradation of HA cryogels to mediate G‐CSF release and enhance neutrophil recovery. As frequent bleeding to measure serum G‐CSF concentrations is challenging in post‐HSCT mice, we assessed G‐CSF release from HA cryogels by labeling G‐CSF with Cy5 and measuring the signal at the site of HA cryogel injection using IVIS microscopy. 1 μg of Cy5‐labeled G‐CSF (Cy5 G‐CSF) was encapsulated in HA cryogels and one cryogel was injected either in 1‐day post‐HSCT or in nonirradiated B6 mice. Encapsulated Cy5 G‐CSF was quantified using IVIS and normalized to the initial 8‐h timepoint fluorescence signal (Figure 5a). Cy5 G‐CSF release, assessed by fluorescence attenuation, from nonirradiated mice proceeded in a sustained manner immediately post‐injection with over 80% released after approximately 12‐days post‐injection. In post‐HSCT mice, 20% Cy5 G‐CSF released after approximately 12‐days post‐injection and subsequently released in a sustained manner (Figure 5b). The time to 50% fluorescence intensity in nonirradiated mice was 5.9 ± 3.0 days compared to 15.5 ± 5.9 days in post‐HSCT mice (Figure 5c). To approximate G‐CSF pharmacokinetics (PK) in the blood, the release profile of G‐CSF from HA cryogels in post‐HSCT mice was modeled as a piecewise function (Note S2). We then sought to assess the effect of G‐CSF delivery on peripheral blood neutrophil recovery and acceleration of Cy5‐HA cryogel degradation. We compared mice receiving either two blank Cy5‐HA cryogels or two G‐CSF‐encapsulated HA‐cryogels and, as a positive control, we included mice with blank Cy5‐HA cryogels injected systemically with 2 μg pegylated (PEG) G‐CSF (Figure 5d), corresponding to the clinical‐equivalent dose for mice.^33^, ^34^ Mice were bled at pre‐determined timepoints, and peripheral blood neutrophil concentration, quantified by flow cytometry, was consistently higher when G‐CSF from Cy5‐HA cryogels was delivered, and comparable with PEG G‐CSF treatment than in mice which received blank Cy5‐HA cryogels (Figure 5e). Moreover, Cy5‐HA cryogel degradation was accelerated with G‐CSF or PEG G‐CSF treatment (Figure 5f; Figure S8). These results support that G‐CSF release from HA cryogels can improve neutrophil recovery in lethally radiated mice and Cy5‐HA cryogel degradation may simultaneously be used as an indicator of functional neutrophil recovery.

## 3 DISCUSSION

Here, we demonstrate that an immune responsive biodegradable HA cryogel scaffold provides sustained G‐CSF release and accelerates post‐HSCT neutrophil recovery in mice which, in turn, accelerates HA cryogel degradation in vivo. Harnessing post‐HSCT immune deficiency to sustain G‐CSF release is distinct conceptually from other methods of drug delivery. It is well established that immune cells sense implanted materials as non‐self and mount a well‐characterized sequential response to isolate the implant in a fibrous capsule.^35^, ^36^, ^37^ In this work, we observed neutrophil infiltration during the acute stages of inflammation and show them to be key mediators in HA cryogel degradation. Our finding is consistent with prior reports that have supported neutrophils as key mediators of shaping the early implant microenvironment and for in vivo destruction of implanted polymeric materials by neutrophil‐derived oxidants.^38^, ^39^, ^40^ The finding of primarily myeloid‐lineage immune cell populations within the HA cryogel is consistent with previous observations of cell infiltration occurring within scaffolds of a similar composition.^41^, ^42^ We demonstrate that the encapsulation and release of G‐CSF from the polymer scaffold mediated recovery of neutrophils in the peripheral blood, significantly faster than control mice receiving blank HA cryogels and comparable to pegylated GCSF, which accelerated HA cryogel degradation.

HA was selected as the primary constituent polymer as it is ubiquitous in the extracellular matrix and has a long history of clinical use as a biodegradable material in a range of biomedical applications.^43^, ^44^, ^45^, ^46^, ^47^, ^48^ In this work, commercially purchased HA was derivatized with bioorthogonal Tz and Nb groups to facilitate crosslinking without the need for external energy input or addition of external agents such as stabilizers and catalysts,^49^, ^50^ which can make it challenging to purify the final product. The use of HA‐Tz and HA‐Nb also facilitated cryogelation at a slower rate, compared to free‐radical polymerization methods, and consequently provided enhanced control over the crosslinking process.^51^, ^52^ Moreover, other common cross‐linking strategies that directly target the carboxylic acid or hydroxyl side chains groups and unreacted agents may inadvertently react with encapsulated proteins.^53^, ^54^, ^55^, ^56^ Further, Tz can be quantified spectroscopically and the DOS was readily assessed.^57^, ^58^

Degradation of HA can be mediated by both enzymatic and nonenzymatic reactions. Enzymatic degradation is mediated by hyaluronidases, a class of enzymes that degrade predominantly HA and are widely distributed in mammalian tissues in the extracellular space, on the cell surface of stromal cells and intracellularly in lysosomal compartments.^59^ Endogenous nonenzymatic reactions have been demonstrated to be mediated predominately by reactive oxygen species for which neutrophils are a major source, and which lack hyaluronidase.^60^, ^61^ Consistent with prior work, our results show that DOS affected the rate of HA cryogel degradation by enzymatic cleavage in vitro.^22^, ^62^ On the other hand, the paradoxical observation that DOS did not affect in vivo degradation is also consistent with prior work that has demonstrated that partial degradation of HA by nonenzymatic means in vivo overcomes steric factors which might otherwise hinder enzymatic access to HA and, in our work, facilitated equalization of the in vivo degradation rate of low‐ and high‐ DOS HA cryogels.^62^

Our results support that activated neutrophils mediate degradation of HA cryogels in vivo, consistent with past reports of the role of reactive oxygen species from activated neutrophils in mediating HA degradation^23^, ^60^, ^63^, ^64^ and of neutrophils in the acute phase of the foreign body response,^35^, ^36^ which further clarifies how immune deficiency impacts the rate of degradation.^9^, ^65^ We found that while despite successfully depleting neutrophils in the peripheral blood, antibody‐based depletion did not achieve a similar depletion of infiltrating neutrophils in HA cryogels and degradation was unaffected in B6 mice. In NSG mice, which have defective adaptive and innate immune cells, Cy5‐HA cryogels degraded minimally over 3 months and neutrophil infiltration into the HA cryogel was not sustained. The observation is consistent with the well‐documented lack of adaptive immune cells, impaired innate immune cell subsets (e.g., macrophages) and a lack of a functional complement system which affects the activation of neutrophils in these mice.^66^, ^67^, ^68^, ^69^ We expanded upon these results by quantifying Cy5‐HA cryogel degradation in gp91^phox−^ mice, which are on the B6 background, but gp91^phox−^ neutrophils in affected hemizygous male mice lack superoxide production.^29^, ^30^ The functional deficiency of neutrophils in gp91^phox−^ is similar to the clinical observations of defective respiratory burst and phagocytosis affecting neutrophils in chronic granulomatous disease, in which there are normal neutrophil counts but impaired oxidative killing.^29^ In these mice, the absence of appreciable degradation of Cy5‐HA cryogels provides additional support for the key role of functional neutrophils in facilitating degradation.

The key role of functional neutrophils in HA cryogel degradation was further validated in post‐HSCT mice, modeling transient innate immune deficiency. Unlike antibody‐based depletion, irradiating mice achieves full depletion of innate and adaptive immune cells. Neutrophils predominate the earliest immune cells that reconstitute^70^ and post‐HSCT respiratory burst and phagocytic activity of neutrophils is generally decreased in humans, modeled by gp91^phox−^ mice, and underscores the importance of qualitatively assessing functionality of neutrophils.^71^ We found that Cy5‐HA cryogel degradation was delayed until neutrophil infiltration into Cy5‐HA cryogels recovered, further supporting the role of neutrophils in mediating HA degradation and the immune responsive behavior of HA cryogels. These results are consistent with past reports in which rapid neutrophil infiltration and activation have been identified as one of the earliest cellular events of the foreign body response.^2^, ^72^ In contrast, we show that OxAlg cryogels, which have hydrolytically labile groups but are not a substrate for endogenous enzymes, degrade rapidly in vivo at similar rates in immune competent and post‐HSCT mice.^31^, ^32^, ^73^ These observations characterizing the importance of neutrophils in degradation support the unique immune responsiveness of HA cryogels.

Similar to PEG‐G‐CSF, we demonstrate the effect of G‐CSF release from HA cryogels is neutrophil‐dependent,^74^ and therefore might be characterized as self‐regulating. However, in contrast to PEG‐G‐CSF, G‐CSF delivery from HA cryogels avoids the potential of pre‐existing or induced anti‐PEG antibody (APA)‐mediated rapid clearance.^75^, ^76^ In immune competent mice, it has been demonstrated that the administration of PEG‐G‐CSF at a clinically‐relevant single dose elicits anti‐PEG IgM antibodies in a dose‐dependent manner which subsequently accelerates clearance of a second PEG‐G‐CSF dose via an anti‐PEG IgM‐mediated complement activation.^21^ De novo anti‐PEG antibody induction may not require T cell activation^77^and therefore could also be induced in post‐HSCT immunodeficient hosts. PEG G‐CSF may therefore be less effective with pre‐existing or induced APA.^78^

Therapy‐induced neutropenia substantially limits the applicability of therapies that could be life‐saving. HA cryogels not only deliver G‐CSF in a sustained manner to enhance neutrophil regeneration, while avoiding the potential of APA‐mediated enhanced clearance, but also show a responsive degradation behavior. Collectively, our findings support that the HA cryogels might be leveraged to enhance and functionally assess neutrophil functionality and aid in treatment‐related decisions for recipients of myelosuppressive therapy.

## 4 METHODS

### 4.1 General methods and statistics

Sample sizes for animal studies were based on prior work without use of additional statistical estimations. Results were analyzed where indicated using student's *t*‐test and two‐way ANOVA with Bonferroni's test using Graphpad Prism software. Mixed‐model linear regression was conducted using IBM SPSS statistical package. Alphanumeric coding was used in blinding for pathology samples and cell counting.

### 4.2 Chemicals

Sodium hyaluronate (MW 1.5–2.2 MDa, Pharma Grade 150, lot: 18011K) and sodium alginate (MW ~250 kDa, Pronova UP MVG) were purchased from NovaMatrix. (2‐morpholinoethanesulfonic acid (MES), sodium chloride (NaCl), sodium hydroxide (NaOH), *N*‐hydroxysuccinimide (NHS), 1‐ethyl‐3‐(3‐dimethylaminopropyl)‐carbodiimide hydrochloride (EDC), sodium periodate (311448) and ammonia borane (AB) complex (682098) were purchased from Sigma‐Aldrich. (4‐(1,2,4,5‐tetrzain‐3‐yl)phenyl)methanamine (tetrazine amine) was purchased from Kerafast (FCC659, lot: 2014). 1‐bicyclo[2.2.1]hept‐5‐en‐2‐ylmethanamine (norbornene amine) was purchased from Matrix Scientific (# 038023, lot: M15S). Cy5‐tetrazine amine was purchased from Lumiprobe (lot: 9D2FH). 1 kDa molecular weight cutoff (MWCO) mPES membrane was purchased from Spectrum (S02‐E001‐05‐N).

### 4.3 Derivatization of HA

Tetrazine functionalized HA (HA‐Tz) or norbornene functionalized HA (HA‐Nb) were prepared by reacting tetrazine amine or norbornene amine to HA using EDC/NHS carbodiimide chemistry. Sodium hyaluronate was dissolved in a buffer solution (0.75% wt/vol, pH ~ 6.5) of 100 mM MES buffer. NHS and EDC were added to the mixture to activate the carboxylic acid groups on the HA backbone followed by either tetrazine amine or norbornene amine. HA was assumed to be 1.8 MDa for purposes of conjugation reactions. To synthesize 7% DOS HA‐Tz (high‐DOS), the molar ratios of HA:EDC:NHS:tetrazine are 1:25000:25000:2500. To synthesize 0.8% DOS HA‐Tz (low‐DOS), the molar ratios of HA:EDC:NHS:tetrazine are 1:2860:2860:286. To synthesize HA‐Nb, the molar ratios of HA:EDC:NHS:norbornene are 1:25000:25000:2500. Each reaction was stirred at room temperature for 24 h and transferred to a 12,000 Da MW cutoff dialysis sack (Sigma Aldrich) and dialyzed in 4 L of NaCl solutions of decreasing molarity (0.125 M, 0.100 M, 0.075 M, 0.050 M, 0.025 M, 0 M, 0 M, 0 M, 0 M) for 8 h per solution. After dialysis, solutions containing HA‐Tz or HA‐Nb were frozen overnight and lyophilized (Labconco Freezone 4.5) for 48 h. Cy5 conjugated HA‐Nb (Cy5‐HA‐Nb) was synthesized following a previously described technique with some modifications.^79^ 0.8 mg of Cy5‐Tz was reacted with 100 mg of HA‐Nb at 0.2 wt/vol in DI water for 24 h at 37°C and purified by dialysis in DI water using a 12,000 Da MW cutoff dialysis sack for 48 h. Dialysis water bath was changed every ~8 h. The Cy5‐HA‐Nb solution was then frozen overnight and lyophilized for 48 h.

### 4.4 Preparation of oxidized alginate

Alginate was oxidized by mixing a 1% wt/vol solution of sodium alginate in DI water with an aqueous solution of 23 mM sodium periodate (Sigma Aldrich) to achieve a 1:586 molar ratio of alginate: periodate. The reaction was stirred in the dark at room temperature overnight. Sodium chloride (1.8 grams/gram of alginate) was added to solution to achieve a 0.3 M solution, followed by purification via tangential flow filtration (TFF) using a mPES 1 kDa molecular weight cutoff (MWCO) membrane (Spectrum) and sequential solvent exchanges with 0.15 M − 0.10 M − 0.05 M and 0.0 M sodium chloride in DI water. The resulting solution was treated with ammonia borane (AB) complex (Sigma Aldrich) at 1:4 alginate:AB molar ratio and stirred at room temperature overnight. Sodium chloride (1.8 grams/gram of alginate) was added to solution to achieve a 0.3 M solution, followed by purification via TFF using a 1 kDa MWCO mPES membrane and sequential solvent exchanges with 0.15 M − 0.10 M − 0.05 M and 0.0 M sodium chloride in DI water. The resulting solution was lyophilized to dryness.

### 4.5 Derivatization of oxidized alginate

To synthesize tetrazine and norbornene functionalized oxidized alginate (OxAlg‐Tz, OxAlg‐Nb respectively), oxidized alginate, prepared as described above, was solubilized in 0.1 M MES buffer,0.3 M sodium chloride, pH 6.5 at 1%wt/vol. NHS and EDC were added to the mixture followed by either tetrazine or norbornene. The molar ratio of oxidized alginate:NHS:EDC:tetrazine or norbornene was 1:5000:5000:1000. The reaction is stirred in the dark at room temperature overnight. The resulting solution is centrifuged at 4700 rpm for 15 min and filtered through a 0.2‐micron filter. The solution is purified via TFF using a mPES 1 kDa molecular weight cutoff (MWCO) membrane and sequential solvent exchanges with 0.15 M − 0.10 M − 0.05 M and 0.0 M sodium chloride in DI water. The purified solution is treated with activated charcoal (1 gram/gram of alginate) for 20 min at room temperature. The slurry is filtered through 0.2‐micron filter and the filtrate is lyophilized to dryness.

### 4.6 Endotoxin testing

Endotoxin testing of high‐DOS HA‐Tz and Cy5‐HA‐Nb were conducted using a commercially available endotoxin testing kit (88282, Thermo Fisher Scientific, lot: VH310729) and following manufacturer's instructions. High‐DOS HA‐Tz and Cy5‐HA‐Nb were solubilized at 0.6 wt% in endotoxin free water and samples were tested in technical triplicates. To calculate endotoxin content of a single HA cryogel, the EU/ml concentration for high‐DOS HA‐Tz and Cy5‐HA‐Nb were divided by 2 (relative concentrations of HA‐Tz and HA‐Nb in HA cryogels are 0.3 wt%) and multiplied by 0.03 (30 μl of volume per HA cryogel). EU/kg was calculated based on 2 HA cryogels administered into a mouse with an average weight of 20 g.

### 4.7 Cryogel development

We followed a previously described cryogelation method.^79^, ^80^, ^81^ To form cryogels, aqueous solutions of 0.6% wt/vol HA‐Tz and HA‐Nb or OxAlg‐ Tz and OxAlg‐Nb were prepared by dissolving lyophilized polymers into deionized water and left on a rocker at room temperature for a minimum of 8 h to allow for dissolution. The aqueous solutions were then pre‐cooled to 4°C before cross‐linking to slow reaction kinetics. HA‐Tz and HA‐Nb or OxAlg‐ Tz and OxAlg‐Nb solutions were mixed at a 1:1 volume ratio, pipetted into 30 μl Teflon molds which were pre‐cooled to −20°C, and quickly transferred to a −20°C freezer to allow for overnight cryogelation. Synthesis of Cy5‐HA or Cy5‐OxAlg cryogels follows the same protocol as above, substituting Cy5‐HA‐Nb for HA‐Nb or Cy5‐OxAlg‐Nb for OxAlg‐Nb.

### 4.8 Pore size analysis of HA cryogels

For SEM, frozen HA cryogels were lyophilized for 24 h and in a petri dish. Lyophilized HA cryogels were adhered onto sample stubs using carbon tape and coated with iridium in a sputter coater. Samples were imaged using secondary electron detection on a FEI Quanta 250 field emission SEM in the Nano3 user facility at UC San Diego. Fluorescence images of Cy5‐HA cryogels were acquired using a Leica SP8 All experiments were performed at the UC San Diego School of Medicine Microscopy Core. Pore size quantification of SEM images and relative distribution of pore sizes of confocal images was doing using FIJI image processing package.^82^

### 4.9 HA cryogel pore‐interconnectedness analysis

Cy5‐HA cryogels were synthesized with low‐ and high‐DOS HA‐Tz and incubated in 1 ml of FITC‐labeled 10 μM diameter melamine resin micro particles (Sigma Aldrich) at 0.29 mg/ml concentration on a rocker at room temperature overnight. Fluorescence images of Cy5‐HA cryogels with FITC‐labeled microparticles were acquired using a Leica SP8 confocal. Interconnectedness of the HA cryogels was determined by generating 3D renderings of confocal z‐stacks using FIJI imaging processing package and assessing fluorescence intensity of both the Cy5 and FITC channels with depth starting from the top of the HA cryogel. To determine the effect of injection on pore interconnectedness, HA cryogels were injected through a 16G needle prior to incubation in FITC‐labeled microparticle solution. All experiments were performed at the UC San Diego School of Medicine Microscopy Core.

### 4.10 In vitro degradation of Cy5‐HA cryogels

Cy5‐HA cryogels synthesized with low‐ and high‐DOS HA‐Tz and placed into individual 1.5 ml microcentrifuge tubes (Thermo Scientific) with 1 ml of 100 U/ml Hyaluronidase from sheep testes Type II (HYAL2, H2126, Sigma Aldrich, lot: SLBZ9984) in 1× PBS. Degradation studies were conducted in tissue culture incubators at 37°C. Supernatant from samples were collected every 24–72 h by centrifuging the samples at room temperature at 2000G for 5 min and removing 0.9 ml of supernatant. Cy5‐HA cryogels were resuspended by adding 0.9 ml of freshly made 100 U/ml HYAL2 in 1× PBS. Fluorescence measurements were conducted using a Nanodrop 2000 Spectrophotometer (Thermo Fisher Scientific) and these values were normalized to sum of the fluorescence values over the course of the experiment. All experiments were performed at UC San Diego.

### 4.11 In vitro degradation of Cy5‐OxAlg cryogels

Cy5‐OxAlg cryogels were placed into individual 1.5 ml microcentrifuge tube with 1 ml of 1× PBS. Degradation studies were conducted in tissue culture incubators at 37°C. Supernatant from samples were collected every 24–72 h by removing visible Cy5‐OxAlg cryogel material with tweezers and transferring to new 1.5 ml microcentrifuge tube with 1 ml of 1× PBS. Fluorescence measurements were conducted using a Nanodrop 2000 Spectrophotometer and these values were normalized to sum of the fluorescence values over the course of the experiment. All experiments were performed at UC San Diego.

### 4.12 In vivo mouse experiments

All animal work was conducted at the Moores Cancer Center vivarium at UC San Diego, except NSG mouse IVIS imaging experiments, which were conducted at the Harvard Biological Research Infrastructure vivarium at Harvard University and approved by the respective Institutional Animal Care and Use Committee (IACUC). All animal experiments followed the National Institutes of Health guidelines and relevant AALAC‐approved procedures. Female C57BL/6J (B6, Jax # 000664) and NOD.Cg‐*Prkdc* ^*scid*^ *Il2rg* ^*tm1Wjl*^/SzJ (NSG, Jax # 005557) mice were 6–8 weeks at the start of the experiments. Male B6.129S‐Cybb^tm1Din^ (gp91^phox−^, Jax # 002365) mice were 6–8 weeks old at the start of experiments. All mice in each experiment were age matched and no randomization was performed. The pre‐established criteria for animal omission were failure to inject the desired cell dose in transplanted mice and death due to transplant failure. Health concerns unrelated to the study (e.g., malocclusion) and known mouse‐strain specific conditions that affected measurements (e.g., severe dermatitis and skin hyperpigmentation in B6 mice) were criteria for omission.

### 4.13 Immune depletion in mice

Neutrophil depletion in B6 mice was achieved by following the previously established protocol.^28^, ^83^ Briefly, 25 μl of anti‐mouse Ly6G antibody (1A8, Bio X Cell, lot: 737719A2)) was administered i.p. every day for the first week. Concurrently, 50 μl of anti‐rat κ immunoglobulin light chain antibody (MAR 18.5, Bio X Cell, lot: 752020 J2) was administered every other day starting on the second day of depletion. After 1 week, the dose of the anti‐mouse Ly6G antibody was increased to 50 μl. Macrophage depletion in B6 mice was induced by i.p. administration of 100 μl of clodronate liposomes (Liposoma, lot: C44J0920) every 3‐days. B cell lineage depletion in B6 mice was induced by i.p. administration of 400 μg of anti‐mouse B220/CD45R antibody (RA3.31/6.1, Bio X Cell, lot: 754420 N1) once every 3‐days. T cell lineage depletion in B6 mice was induced by i.p. administration of 400 μg dose of anti‐mouse CD4 antibody (GK1.5, Bio X Cell, lot: 728319 M2) and 400 μg dose of anti‐mouse CD8α antibody (2.43, Bio X Cell, lot:732020F1) once every 3‐days. For all lineage depletion models, mice received intraperitoneal injections of 0.1 ml (400 μg) of antibodies or 0.1 ml of clodronate liposome solution 3 days before subcutaneous HA cryogel or Cy5‐HA cryogel injection. Depletion started 3‐days prior to Cy5‐HA cryogel administration to mice and continued until complete cryogel degradation or until mice were euthanized and cryogels retrieved for analysis. All experiments were performed at the Moores Cancer Center vivarium at UC San Diego.

### 4.14 Transplant models

Irradiations were performed with a Cesium‐137 gamma‐radiation source irradiator (J.L. Shepherd & Co.). Syngeneic HSCT (B6 recipients) consisted of 1 dose of 1000 cGy + 1 × 10^5^ lineage‐depleted bone marrow cells from syngeneic B6 donors. Bone marrow cells for transplantation (from donors) or analysis were harvested by crushing all limbs with a mortar and pestle, diluted in 1× PBS, filtering the tissue homogenate through a 70 μm mesh and preparing a single‐cell suspension by passing the cells in the flowthrough once through a 20‐gauge needle. Total cellularity was determined by counting cells using a hemacytometer. Bone marrow cells were depleted of immune cells (expressing CD3ε, CD45R/B220, Ter‐119, CD11b, or Gr‐1) by magnetic selection using a Mouse Hematopoietic Progenitor Cell Enrichment Set (BD Biosciences # 558451, lot: 0114777). To confirm depletion, we incubated cells with a mix of Pacific Blue‐conjugated lineage specific antibodies (antibodies to CD3, NK1.1, Gr‐1, CD11b, CD19, CD4 and CD8) and with Sca‐1 and cKit‐specific antibodies for surface staining and quantification of Lineage^−^ fraction of cells, which were ≥ 87% lineage depleted. Subsequently, cells were suspended in 100 μl of sterile 1× PBS and administered to anesthetized mice via a single retroorbital injection. All experiments were performed at the Moores Cancer Animal Facility at UC San Diego Health. All flow cytometry experiments were performed using an Attune® NxT Acoustic Focusing cytometer analyzer (A24858) at UC San Diego.

### 4.15 Subcutaneous cryogel administration

While mice were anesthetized, a subset received a subcutaneous injection of HA cryogel or OxAlg cryogel, which was suspended in 200 μl of sterile 1× PBS, into the dorsal flank by means of a 16G needle positioned approximately midway between the hind‐ and forelimbs. The site of injection was shaved and wiped with a sterile alcohol pad prior to gel injection.

### 4.16 In vivo degradation

In vivo Cy5‐HA cryogel degradation was performed with Cy5‐HA cryogels synthesized with low‐ and high‐DOS Tz‐HA in untreated B6 mice, immune deficient B6 mice, NSG mice, and gp91^phox−^ mice. In vivo Cy5 OxAlg cryogel degradation was performed in nonirradiated, non‐transplanted B6 mice and B6 mice post‐HSCT. In all cases, cryogels were administered into the dorsal flank of an anesthetized mouse and the fluorescent intensity of the Cy5‐HA cryogel was quantified using an IVIS spectrometer (PerkinElmer) at predetermined timepoints and analyzed using LivingImage software (PerkinElmer). At each timepoint, mice were anesthetized and the area around the subcutaneous cryogel was shaved to reduce fluorescence signal attenuation. Fluorescence radiant efficiency, the ratio of fluorescence emission to excitation, was measured longitudinally as a metric to quantify fluorescence from subcutaneous cryogels. These values were normalized to the measured signal on day 3. All experiments were performed at the Moores Cancer Microscopy Core Facility at UC San Diego Health, with the exception of NSG mouse in vivo degradation experiments, which were performed at the Harvard Biology Research Infrastructure vivarium using an IVIS spectrometer (PerkinElmer).

### 4.17 Flow cytometry analysis

Anti‐mouse antibodies to CD45 (30‐F11, lot: B280746), CD11b (M1/70, lot: B322056), CD4 (RM4‐5, lot: B240051), CD8α (53–6.7, lot: B266721), B220 (RA3‐6B2, lot: B298555), Ly6‐G/Gr‐1 (1A8, lot: B259670), lineage cocktail (17A2/RB6‐8C5/RA3‐6B2/Ter‐119/M1/70, lot: B266946), Ly‐6A/E/Sca‐1 (D7, lot: B249343), and CD117/cKit (2B8, lot: B272462) were purchased from Biolegend. Anti‐mouse F4/80 (BM8, lot: 2229150) and was purchased from eBioscience. All cells were gated based on forward and side scatter characteristics to limit debris, including dead cells. AnnexinV (Biolegend, lot: B300974) stain was used to separate live and dead cells. Antibodies were diluted according to the manufacturer's suggestions. Cells were gated based on fluorescence‐minus‐one controls, and the frequencies of cells staining positive for each marker were recorded. To quantify T cells, B cells, monocytes, and neutrophils in peripheral blood, blood was first collected from the tail vein of mice into EDTA coated tubes (BD). Samples then underwent lysis of red blood cells and were stained with appropriate antibodies corresponding to cell populations of interest. To quantify infiltrating immune cells within Cy5‐HA cryogels, mice were sacrificed, cryogels removed, and HA cryogels crushed against a 70‐micron filter screen before antibody staining. Absolute numbers of cells were calculated using flow cytometry frequency. Flow cytometry was analyzed using FlowJo (BD) software. All flow cytometry experiments were performed using a Attune® NxT Acoustic Focusing cytometer analyzer (A24858) at UC San Diego.

### 4.18 Histology

After euthanasia, HA cryogels were explanted and fixed in 4% paraformaldehyde (PFA) for 24 h. The fixed HA cryogels were then transferred to 70% ethanol solution. Samples were routinely processed and sections (5 μm) were stained and digitized using an Aperio AT2 Automated Digital Whole Slide Scanner by the Tissue Technology Shared Resource at the Moores Cancer Center at UC San Diego Health. Digital slides were rendered in QuPath and positive cell detection was used to quantify the total number of mononuclear cells within each image. Quantification of mononuclear cell density was determined for each histological section.

### 4.19 Immunohistochemistry

Paraffin embedded HA cryogel sections were baked at 60°C for 1 h. Tissues were then rehydrated through successive washes (3× xylene, 2× 100% ethanol, 2× 95% ethanol, 2× 70% ethanol, di‐water). After rehydration, antigen retrieval was conducted using Unmasking solution (Citrate based, pH 6) (Vector Laboratories, H‐3300) at 95°C for 30 min. Staining was performed using Intellipath Automated IHC Stainer (Biocare). A peroxidase block, Bloxall (Vector Laboratories, SP‐6000) was performed for 10 min, followed by 2× washes in 1× tris‐buffered saline with 0.1% Tween 20 (TBST, Sigma Aldrich), and a blocking step using 3% Donkey Serum for 10 min. Samples were stained using anti‐Ly6G primary antibody (Rabbit, Cell Signaling Technology, 87048S) at 1:100 concentration for 1 h. Samples were washed twice in TBST and anti‐rabbit HRP polymer (Cell IDX, 2HR‐100) was added for 30 min. Samples were washed twice in TBST and DAB (brown) Chromogen (VWR, 95041‐478) was added for 5 min. This was followed by 2× washes in di‐water, 5‐min incubation with Mayer's Hematoxylin (Sigma, 51275), 2× washes in TBST, and 1× wash in di‐water. Mounting was performed using a xylene‐based mountant. IHC was performed by the Tissue Technology Shared Resource at the Moores Cancer Center at UC San Diego Health.

### 4.20 G‐CSF encapsulation

To quantify G‐CSF release from HA cryogels, recombinant human G‐CSF (300–23, Peprotech, lots: 041777 and 041877) was reacted with sulfo‐Cy5 NHS ester (13,320, Lumiprobe, lot 7FM7C) at a 1:250:25 molar ratio of G‐CSF:EDC:sulfo‐Cy5 NHS ester in MES buffer to form Cy5 G‐CSF. Unreacted EDC and sulfo‐Cy5 NHS ester was removed by overnight dialysis on a 10 kDa dialysis membrane. 1 μg of Cy5 G‐CSF was added to 0.6% wt/vol HA‐Tz solution before mixing with HA‐Nb and cryogelation as described above. To track Cy5‐HA cryogel degradation in mice which received G‐CSF loaded Cy5‐HA cryogels, the same protocol is followed substituting G‐CSF for Cy5 G‐CSF and Cy5‐HA‐Nb for HA‐Nb.

### 4.21 Neutrophil reconstitution models

Mice were irradiated and administered an autologous HSCT as described above. PEG G‐CSF (MBS355608, MyBioSource, lot: R15/2020 J) or G‐CSF was injected i.p. Cy5‐HA cryogel encapsulating G‐CSF was injected subcutaneously as described above, 24 h post‐HSCT. Mice were bled at predetermined timepoints and relevant immune subsets were stained for analysis by flow cytometry.

## AUTHOR CONTRIBUTIONS

**Matthew Kerr:** Conceptualization (lead); data curation (lead); formal analysis (lead); investigation (lead); methodology (lead); resources (lead); validation (lead); visualization (lead); writing – original draft (lead); writing – review and editing (lead). **David McBride:** Investigation (supporting); methodology (supporting); writing – review and editing (supporting). **Wade Johnson:** Investigation (supporting); writing – review and editing (supporting). **Arun Chumber:** Investigation (supporting); writing – review and editing (supporting). **Alexander Najibi:** Investigation (supporting); writing – review and editing (supporting). **Bo Ri Seo:** Investigation (supporting); writing – review and editing (supporting). **Alexander Stafford:** Resources (supporting). **David Scadden:** Funding acquisition (supporting). **David Mooney:** Conceptualization (equal); funding acquisition (supporting); writing – review and editing (supporting). **Nisarg Shah:** Conceptualization (equal); funding acquisition (lead); project administration (lead); supervision (lead); writing – original draft (supporting); writing – review and editing (supporting).

## CONFLICT OF INTERESTS

Matthew D. Kerr, David J. Mooney, and Nisarg J. Shah are named inventors on US Provisional Patent Application No. 63/110,528.

## Supporting information


**Figure S1** Extended materials characterization of HA cryogels
**Figure S2** Extended characterization of Cy5‐HA cryogel degradation
**Figure S3** Extended characterization of Cy5‐HA cryogel degradation in immunodeficient mice
**Figure S4** Extended histomorphometric analysis of Cy5‐HA cryogels retrieved from T and B cell depleted mice
**Figure S5** Extended analysis of myeloid cell infiltration of Cy5‐HA cryogels retrieved from immunodeficient mice
**Figure S6** Extended immunohistochemical staining of Cy5‐HA cryogels retrieved from untreated B6 and NSG mice
**Figure S7** Extended quantification of post‐HSCT HA cryogel degradation
**Figure S8** Extended characterization of peripheral blood neutrophil reconstitution
Table S1
Table S2

**Appendix S1**: Supplementary figure captions and notes

Movie S1

## Data Availability

The datasets generated during and/or analyzed during the current study are available from the corresponding authors on reasonable request.

## References

[1] Mócsai A . Diverse novel functions of neutrophils in immunity, inflammation, and beyond. J Exp Med. 2013;210:1283‐1299. 23825232 10.1084/jem.20122220 PMC3698517

[2] Anderson JM , Rodriguez A , Chang DT . Foreign body reaction to biomaterials. Semin Immunol. 2008;20:86‐100. 18162407 10.1016/j.smim.2007.11.004 PMC2327202

[3] Wang J , Hossain M , Thanabalasuriar A , Gunzer M , Meininger C , Kubes P . Visualizing the function and fate of neutrophils in sterile injury and repair. Science. 2017;358:111‐116. 28983053 10.1126/science.aam9690

[4] Lakshman R , Finn A . Neutrophil disorders and their management. J Clin Pathol. 2001;54:7‐19. 11271792 10.1136/jcp.54.1.7 PMC1731272

[5] Crawford J , Dale DC , Lyman GH . Chemotherapy‐induced neutropenia: risks, consequences, and new directions for its management. Cancer. 2004;100:228‐237. 14716755 10.1002/cncr.11882

[6] Gil L , Poplawski D , Mol A , Nowicki A , Schneider A , Komarnicki M . Neutropenic enterocolitis after high‐dose chemotherapy and autologous stem cell transplantation: incidence, risk factors, and outcome. Transpl Infect Dis. 2013;15:1‐7. 22862907 10.1111/j.1399-3062.2012.00777.x

[7] Ley K , Hoffman HM , Kubes P , et al. Neutrophils: new insights and open questions. Science Immunol. 2018;3(30):eaat4579. doi:10.1126/sciimmunol.aat4579 30530726

[8] Çelebi H , Akan H , Akçağlayan E , Üstün C , Arat M . Febrile neutropenia in allogeneic and autologous peripheral blood stem cell transplantation and conventional chemotherapy for malignancies. Bone Marrow Transplantation. 2000;26(2):211‐214. doi:10.1038/sj.bmt.1702503 10918433

[9] Sadtler K , Allen BW , Estrellas K , Housseau F , Pardoll DM , Elisseeff JH . The scaffold immune microenvironment: biomaterial‐mediated immune polarization in traumatic and nontraumatic applications. Tissue Eng Part A. 2017;23:1044‐1053. 27736323 10.1089/ten.tea.2016.0304 PMC6436021

[10] Balletto E , Mikulska M . Bacterial infections in hematopoietic stem cell transplant recipients. Meditere J Hematol Infect Dis. 2015;7:e2015045. 10.4084/MJHID.2015.045 PMC4500472 26185610

[11] Stoma I , Karpov I , Milanovich N , Uss A , Iskrov I . Risk factors for mortality in patients with bloodstream infections during the pre‐engraftment period after hematopoietic stem cell transplantation. Blood Res. 2016;51:102‐106. 27382554 10.5045/br.2016.51.2.102 PMC4931927

[12] Matsushima H , Geng S , Lu R , et al. Neutrophil differentiation into a unique hybrid population exhibiting dual phenotype and functionality of neutrophils and dendritic cells. Blood. 2013;121:1677‐1689. 23305731 10.1182/blood-2012-07-445189 PMC3591793

[13] Weiskopf K , Schnorr PJ , Pang WW , et al. Myeloid cell origins, differentiation, and clinical implications. Microbiol Spectr. 2016;4(5). doi:10.1128/microbiolspec.MCHD-0031-2016 PMC5119546 27763252

[14] Trivedi M , Martinez S , Corringham S , Medley K , Ball ED . Optimal use of G‐CSF administration after hematopoietic SCT. Bone Marrow Transplant. 2009;43:895‐908. 19363527 10.1038/bmt.2009.75

[15] Mehta HM , Malandra M , Corey SJ . G‐CSF and GM‐CSF in neutropenia. J Immunol. 2015;195:1341‐1349. 26254266 10.4049/jimmunol.1500861 PMC4741374

[16] Bajrami B , Zhu H , Kwak HJ , et al. G‐CSF maintains controlled neutrophil mobilization during acute inflammation by negatively regulating CXCR2 signaling. J Exp Med. 2016;213:1999‐2018. 27551153 10.1084/jem.20160393 PMC5030805

[17] Pinto L , Liu Z , Doan Q , Bernal M , Dubois R , Lyman G . Comparison of pegfilgrastim with filgrastim on febrile neutropenia, grade IV neutropenia and bone pain: a meta‐analysis of randomized controlled trials. Curr Med Res Opin. 2007;23:2283‐2295. 17697451 10.1185/030079907X219599

[18] Singh AD , Parmar S , Patel K , et al. Granulocyte colony‐stimulating factor use after autologous peripheral blood stem cell transplantation: comparison of two practices. Biol Blood Marrow Transplant. 2018;24:288‐293. 29061534 10.1016/j.bbmt.2017.10.026 PMC6574227

[19] Ashrafi F , Salmasi M . Comparison of the effects of pegylated granulocyte‐colony stimulating factor and granulocyte‐colony stimulating factor on cytopenia induced by dose‐dense chemotherapy in breast cancer patients. J Res Med Sci. 2018;23:73. 30181755 10.4103/jrms.JRMS_463_17 PMC6116661

[20] Molineux G . The design and development of pegfilgrastim (PEG‐rmetHuG‐CSF, Neulasta). Curr Pharm Des. 2004;10:1235‐1244. 15078138 10.2174/1381612043452613

[21] Elsadek NE , Lila ASA , Emam SE , et al. Pegfilgrastim (PEG‐G‐CSF) induces anti‐PEG IgM in a dose dependent manner and causes the accelerated blood clearance (ABC) phenomenon upon repeated administration in mice. Eur J Pharm Biopharm. 2020;152:56‐62. 32376372 10.1016/j.ejpb.2020.04.026

[22] Girard N , Maingonnat C , Bertrand P , Tilly H , Vannier JP , Delpech B . Human monocytes synthesize hyaluronidase. Br J Haematol. 2002;119:199‐203. 12358926 10.1046/j.1365-2141.2002.03733.x

[23] Soltes L , Mendichi R , Kogan G , Schiller J , Stankovska M , Arnhold J . Degradative action of reactive oxygen species on hyaluronan. Biomacromolecules. 2006;7:659‐668. 16529395 10.1021/bm050867v

[24] Jiang D , Liang J , Noble PW . Hyaluronan as an immune regulator in human diseases. Physiol Rev. 2011;91:221‐264. 21248167 10.1152/physrev.00052.2009 PMC3051404

[25] Ito M , Hiramatsu H , Kobayashi K , et al. NOD/SCID/gamma(c)(null) mouse: an excellent recipient mouse model for engraftment of human cells. Blood. 2002;100:3175‐3182. 12384415 10.1182/blood-2001-12-0207

[26] Malyala P , Singh M . Endotoxin limits in formulations for preclinical research. J Pharm Sci. 2008;97:2041‐2044. 17847072 10.1002/jps.21152

[27] Burdick JA , Chung C , Jia X , Randolph MA , Langer R . Controlled degradation and mechanical behavior of photopolymerized hyaluronic acid networks. Biomacromolecules. 2005;6:386‐391. 15638543 10.1021/bm049508a PMC2678566

[28] Boivin G , Faget J , Ancey PB , et al. Durable and controlled depletion of neutrophils in mice. Nat Commun. 2020;11:2762. 32488020 10.1038/s41467-020-16596-9 PMC7265525

[29] Pollock JD , Williams DA , Gifford MA , et al. Mouse model of X‐linked chronic granulomatous disease, an inherited defect in phagocyte superoxide production. Nat Genet. 1995;9:202‐209. 7719350 10.1038/ng0295-202

[30] Banerjee ER , Henderson WR Jr . Role of T cells in a gp91phox knockout murine model of acute allergic asthma. Allergy Asthma Clin Immunol. 2013;9:6. 23390895 10.1186/1710-1492-9-6 PMC3643823

[31] Bouhadir KH , Lee KY , Alsberg E , Damm KL , Anderson KW , Mooney DJ . Degradation of partially oxidized alginate and its potential application for tissue engineering. Biotechnol Prog. 2001;17:945‐950. 11587588 10.1021/bp010070p

[32] Gao C , Liu M , Chen J , Zhang X . Preparation and controlled degradation of oxidized sodium alginate hydrogel. Polymer Degradation and Stability. 2009;94(9):1405‐1410. doi:10.1016/j.polymdegradstab.2009.05.011

[33] Jagasia MH , Greer JP , Morgan DS , et al. Pegfilgrastim after high‐dose chemotherapy and autologous peripheral blood stem cell transplant: phase II study. Bone Marrow Transplant. 2005;35:1165‐1169. 15880129 10.1038/sj.bmt.1704994

[34] Martino M , Praticò G , Messina G , et al. Pegfilgrastim compared with filgrastim after high‐dose melphalan and autologous hematopoietic peripheral blood stem cell transplantation in multiple myeloma patients. Eur J Haematol. 2006;77:410‐415. 16930141 10.1111/j.1600-0609.2006.00736.x

[35] Barr S , Hill EW , Bayat A . Functional biocompatibility testing of silicone breast implants and a novel classification system based on surface roughness. J Mech Behav Biomed Mater. 2017;75:75‐81. 28697402 10.1016/j.jmbbm.2017.06.030

[36] Carnicer‐Lombarte A , Chen S‐T , Malliaras GG , Barone DG . Foreign body reaction to implanted biomaterials and its impact in nerve neuroprosthetics. Front Bioeng Biotechnol. 2021;9:622524. 33937212 10.3389/fbioe.2021.622524 PMC8081831

[37] Whitaker R , Hernaez‐Estrada B , Hernandez RM , Santos‐Vizcaino E , Spiller KL . Immunomodulatory biomaterials for tissue repair. Chem Rev. 2021;121(18):11305‐11335. 34415742 10.1021/acs.chemrev.0c00895

[38] Sutherland K , Mahoney JR , Coury AJ , Eaton JW . Degradation of biomaterials by phagocyte‐derived oxidants. J Clin Invest. 1993;92:2360‐2367. 8227352 10.1172/JCI116841 PMC288418

[39] Ye Q , Harmsen MC , van Luyn MJ , Bank RA . The relationship between collagen scaffold cross‐linking agents and neutrophils in the foreign body reaction. Biomaterials. 2010;31:9192‐9201. 20828809 10.1016/j.biomaterials.2010.08.049

[40] Labow RS , Meek E , Santerre JP . Neutrophil‐mediated biodegradation of medical implant materials. J Cell Physiol. 2001;186:95‐103. 11147818 10.1002/1097-4652(200101)186:1<95::AID-JCP1008>3.0.CO;2-0

[41] Shah NJ , Najibi AJ , Shih TY , et al. A biomaterial‐based vaccine eliciting durable tumour‐specific responses against acute myeloid leukaemia. Nat Biomed Eng. 2020;4:40‐51. 31937942 10.1038/s41551-019-0503-3

[42] Verbeke CS , Gordo S , Schubert DA , et al. Multicomponent injectable hydrogels for antigen‐specific tolerogenic immune modulation. Adv Healthc Mater. 2017;6(6). doi:10.1002/adhm.201600773 PMC5518671 28116870

[43] Frantz C , Stewart KM , Weaver VM . The extracellular matrix at a glance. J Cell Sci. 2010;123:4195‐4200. 21123617 10.1242/jcs.023820 PMC2995612

[44] Kaderli S , Boulocher C , Pillet E , et al. A novel biocompatible hyaluronic acid‐chitosan hybrid hydrogel for osteoarthrosis therapy. Int J Pharm. 2015;483:158‐168. 25666331 10.1016/j.ijpharm.2015.01.052

[45] Kaderli S , Viguier E , Watrelot‐Virieux D , et al. Efficacy study of two novel hyaluronic acid‐based formulations for viscosupplementation therapy in an early osteoarthrosic rabbit model. Eur J Pharm Biopharm. 2015;96:388‐395. 26369477 10.1016/j.ejpb.2015.09.005

[46] Buffa R , Odstrčilová L , Šedová P , Basarabová I , Novotný J , Velebný V . Conjugates of modified hyaluronic acid with amino compounds for biomedical applications. Carbohydr Polym. 2018;189:273‐279. 29580409 10.1016/j.carbpol.2018.02.048

[47] Ma X , Xu T , Chen W , Qin H , Chi B , Ye Z . Injectable hydrogels based on the hyaluronic acid and poly (gamma‐glutamic acid) for controlled protein delivery. Carbohydr Polym. 2018;179:100‐109. 29111032 10.1016/j.carbpol.2017.09.071

[48] Purcell BP , Lobb D , Charati MB , et al. Injectable and bioresponsive hydrogels for on‐demand matrix metalloproteinase inhibition. Nat Mater. 2014;13:653‐661. 24681647 10.1038/nmat3922 PMC4031269

[49] Barker IA , Hall DJ , Hansell CF , et al. Tetrazine‐norbornene click reactions to functionalize degradable polymers derived from lactide. Macromol Rapid Commun. 2011;32:1362‐1366. 25867900 10.1002/marc.201100324

[50] Hansell CF , Espeel P , Stamenović MM , et al. Additive‐free clicking for polymer functionalization and coupling by tetrazine‐norbornene chemistry. J Am Chem Soc. 2011;133:13828‐13831. 21819063 10.1021/ja203957h

[51] Kennedy S , Bencherif S , Norton D , Weinstock L , Mehta M , Mooney D . Rapid and extensive collapse from electrically responsive macroporous hydrogels. Adv Healthc Mater. 2014;3:500‐507. 24030924 10.1002/adhm.201300260 PMC3954446

[52] Koshy ST , Zhang DKY , Grolman JM , Stafford AG , Mooney DJ . Injectable nanocomposite cryogels for versatile protein drug delivery. Acta Biomater. 2018;65:36‐43. 29128539 10.1016/j.actbio.2017.11.024 PMC5716876

[53] Qin X‐H, Gruber P, Markovic M, et al. Enzymatic synthesis of hyaluronic acid vinyl esters for two‐photon microfabrication of biocompatible and biodegradable hydrogel constructs. Polym. Chem.. 2014;5:(22):6523. –6533. 10.1039/c4py00792a

[54] Chen F , Ni Y , Liu B , et al. Self‐crosslinking and injectable hyaluronic acid/RGD‐functionalized pectin hydrogel for cartilage tissue engineering. Carbohydr Polym. 2017;166:31‐44. 28385238 10.1016/j.carbpol.2017.02.059

[55] Tavsanli B , Okay O . Mechanically strong hyaluronic acid hydrogels with an interpenetrating network structure. Eur Polym J. 2017;94:185‐195.

[56] Pedron S , Pritchard AM , Vincil GA , Andrade B , Zimmerman SC , Harley BAC . Patterning three‐dimensional hydrogel microenvironments using hyperbranched polyglycerols for independent control of mesh size and stiffness. Biomacromolecules. 2017;18:1393‐1400. 28245360 10.1021/acs.biomac.7b00118 PMC5444810

[57] Šečkutė J , Yang J , Devaraj NK . Rapid oligonucleotide‐templated fluorogenic tetrazine ligations. Nucleic Acids Res. 2013;41:e148‐e148. 23775794 10.1093/nar/gkt540 PMC3753649

[58] Johann K , Svatunek D , Seidl C , et al. Tetrazine‐ and trans‐cyclooctene‐functionalised polypept(o)ides for fast bioorthogonal tetrazine ligation. Polym Chem. 2020;11:4396‐4407.

[59] Yamaguchi Y , Yamamoto H , Tobisawa Y , Irie F . TMEM2: a missing link in hyaluronan catabolism identified? Matrix Biol. 2019;78‐79:139‐146. 10.1016/j.matbio.2018.03.020 PMC6314907 29601864

[60] Greenwald RA , Moak SA . Degradation of hyaluronic acid by polymorphonuclear leukocytes. Inflammation. 1986;10:15‐30. 3007353 10.1007/BF00916037

[61] Stern R , Kogan G , Jedrzejas MJ , Šoltés L . The many ways to cleave hyaluronan. Biotechnol Adv. 2007;25:537‐557. 17716848 10.1016/j.biotechadv.2007.07.001

[62] Greenwald RA , Moy WW . Effect of oxygen‐derived free radicals on hyaluronic acid. Arthritis Rheum. 1980;23:455‐463. 6245661 10.1002/art.1780230408

[63] McNeil J , Wiebkin O , Betts W , Cleland L . Depolymerisation products of hyaluronic acid after exposure to oxygen‐derived free radicals. Ann Rheum Dis. 1985;44:780‐789. 4062391 10.1136/ard.44.11.780 PMC1001777

[64] Duan J , Kasper DL . Oxidative depolymerization of polysaccharides by reactive oxygen/nitrogen species. Glycobiology. 2011;21:401‐409. 21030538 10.1093/glycob/cwq171 PMC3055593

[65] Doloff JC , Veiseh O , Vegas AJ , et al. Colony stimulating factor‐1 receptor is a central component of the foreign body response to biomaterial implants in rodents and non‐human primates. Nat Mater. 2017;16:671‐680. 28319612 10.1038/nmat4866 PMC5445003

[66] Shultz LD , Schweitzer PA , Christianson SW , et al. Multiple defects in innate and adaptive immunologic function in NOD/LtSz‐scid mice. J Immunol. 1995;154:180‐191. 7995938

[67] Shultz LD , Lyons BL , Burzenski LM , et al. Human lymphoid and myeloid cell development in NOD/LtSz‐scid IL2R gamma null mice engrafted with mobilized human hemopoietic stem cells. J Immunol. 2005;174:6477‐6489. 15879151 10.4049/jimmunol.174.10.6477

[68] Foreman O , Kavirayani AM , Griffey SM , Reader R , Shultz LD . Opportunistic bacterial infections in breeding colonies of the NSG mouse strain. Vet Pathol. 2011;48:495‐499. 20817888 10.1177/0300985810378282 PMC3101569

[69] Patton JB , Bonne‐Année S , Deckman J , et al. Methylprednisolone acetate induces, and Δ7‐dafachronic acid suppresses, Strongyloides stercoralis hyperinfection in NSG mice. Proc Natl Acad Sci U S A. 2018;115:204‐209. 29203662 10.1073/pnas.1712235114 PMC5776800

[70] Pavlů J , Auner HW , Szydlo RM , et al. Analysis of hematopoietic recovery after autologous transplantation as method of quality control for long‐term progenitor cell cryopreservation. Bone Marrow Transplant. 2017;52:1599‐1601. 28650454 10.1038/bmt.2017.113

[71] Ramaprasad C , Pouch S , Pitrak DL . Neutrophil function after bone marrow and hematopoietic stem cell transplant. Leuk Lymphoma. 2010;51:756‐767. 20350278 10.3109/10428191003695678

[72] Grainger DW . All charged up about implanted biomaterials. Nat Biotechnol. 2013;31:507‐509. 23752436 10.1038/nbt.2600

[73] Freedman BR , Uzun O , Luna NMM , et al. Degradable and removable tough adhesive hydrogels. Adv Mater. 2021;33:2008553. 10.1002/adma.202008553 PMC8764582 33763904

[74] Zamboni WC . Pharmacokinetics of pegfilgrastim. Pharmacotherapy. 2003;23:9S‐14S. 12921217 10.1592/phco.23.9.9s.32888

[75] Zhang P , Sun F , Liu S , Jiang S . Anti‐PEG antibodies in the clinic: current issues and beyond PEGylation. J Control Release. 2016;244:184‐193. 27369864 10.1016/j.jconrel.2016.06.040 PMC5747248

[76] McSweeney MD , Wessler T , Price LSL , et al. A minimal physiologically based pharmacokinetic model that predicts anti‐PEG IgG‐mediated clearance of PEGylated drugs in human and mouse. J Control Release. 2018;284:171‐178. 29879519 10.1016/j.jconrel.2018.06.002 PMC6087483

[77] Ishida T , Wang X , Shimizu T , Nawata K , Kiwada H . PEGylated liposomes elicit an anti‐PEG IgM response in a T cell‐independent manner. J Control Release. 2007;122:349‐355. 17610982 10.1016/j.jconrel.2007.05.015

[78] Yang Q , Jacobs TM , McCallen JD , et al. Analysis of pre‐existing IgG and IgM antibodies against polyethylene glycol (PEG) in the general population. Anal Chem. 2016;88:11804‐11812. 27804292 10.1021/acs.analchem.6b03437 PMC6512330

[79] Koshy ST , Desai RM , Joly P , et al. Click‐crosslinked injectable gelatin hydrogels. Adv Healthc Mater. 2016;5:541‐547. 26806652 10.1002/adhm.201500757 PMC4849477

[80] Shah NJ , Mao AS , Shih TY , et al. An injectable bone marrow‐like scaffold enhances T cell immunity after hematopoietic stem cell transplantation. Nat Biotechnol. 2019;37:293‐302. 30742125 10.1038/s41587-019-0017-2 PMC6636841

[81] Desai RM , Koshy ST , Hilderbrand SA , Mooney DJ , Joshi NS . Versatile click alginate hydrogels crosslinked via tetrazine‐norbornene chemistry. Biomaterials. 2015;50:30‐37. 25736493 10.1016/j.biomaterials.2015.01.048

[82] Schindelin J , Arganda‐Carreras I , Frise E , et al. Fiji: an open‐source platform for biological‐image analysis. Nat Methods. 2012;9:676‐682. 22743772 10.1038/nmeth.2019 PMC3855844

[83] Stackowicz J , Jönsson F , Reber LL . Mouse models and tools for the in vivo study of neutrophils. Front Immunol. 2020;10:3130. 32038641 10.3389/fimmu.2019.03130 PMC6985372

