# Supplementary material for: Immune‐responsive biodegradable scaffolds for enhancing neutrophil regeneration
Source: Bioeng Transl Med. 2022 Apr 19;8(1):e10309. doi: 10.1002/btm2.10309 (PMC9842036; doi:10.1002/btm2.10309)

Supplementary Table 1

Material	Sample 1 (EU/mL)	Sample 2 (EU/mL)	Sample 3 (EU/mL)
HA-Tz	0.332	0.367	0.362
Cy5-HA-Nb	0.227	0.23	0.229
HA Cryogel Average Endotoxin Content	0.00874 EU		
EU/kg (2 HA cryogels/mouse)	0.874 EU/kg		

Supplementary Table 2

Depletion Type	Depletion Agent	Dose, Administration Route, Frequency	Depletion Efficiency
Neutrophil Depletion	Anti-mouse Ly6G antibody (1A8, Bio X Cell)<br>Anti-rat kappa immunoglobulin light chain antibody (MAR 18.5, Bio X Cell)	25μL anti-mouse Ly6G - IP administration - Everyday for 1 week<br>50μL anti-mouse Ly6G - IP administration - Everyday after first week<br>50μL anti-rat kappa immunoglobulin light chain - IP administration - Every other day	Consistent - 98% Depletion of neutrophils in peripheral blood (Supplemental 5b,5c)
Macrophage/Monocyte Depletion	Clodronate Liposomes (Liposoma)	100μL - IP administration - 2x/week	Consistent - 80-95% Depletion of monocytes in peripheral blood (Supplemental 5d, 5e)
T-cell Depletion	Anti-mouse CD4 antibody (GK1.5, Bio X Cell)<br>Anti-mouse CD8α antibody (2.43, Bio X Cell)	400μg anti-mouse CD4 - IP administration - 2x/week<br>400μg anti-mouse CD8α - IP administration - 2x/week	Consistent - 99% Depletion of T-cells in peripheral blood (Supplemental 5f, 5g)
B-cell Depletion	Anti-mouse B220/CD45R antibody (RA3.31, Bio X Cell)	400μg anti-mouse B220/CD45R - IP administration - 2x/week	Transient - 99% Depletion of B-cells in peripheral blood 4 days after start of depletion. Full reconstitution of B-cells by 3 weeks. (Supplemental 5h, 5i)

Supplementary Figure 1

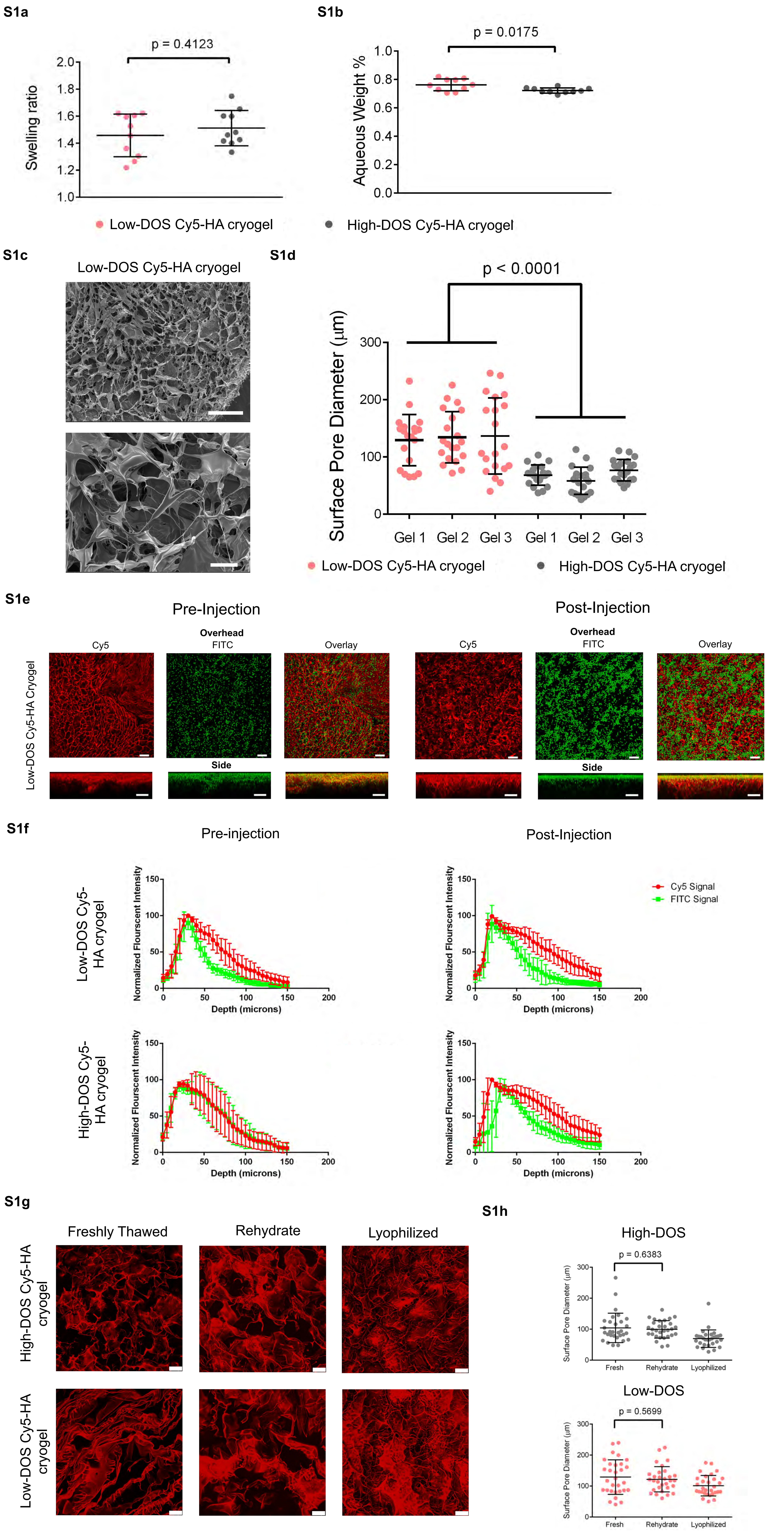

Supplementary Figure 2

S2a

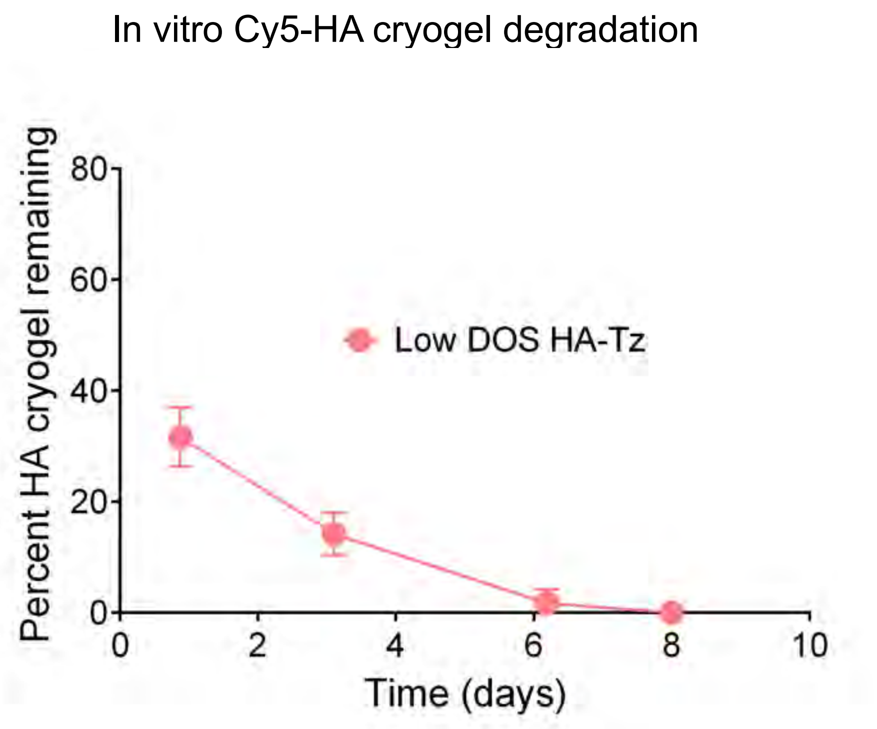

S2b

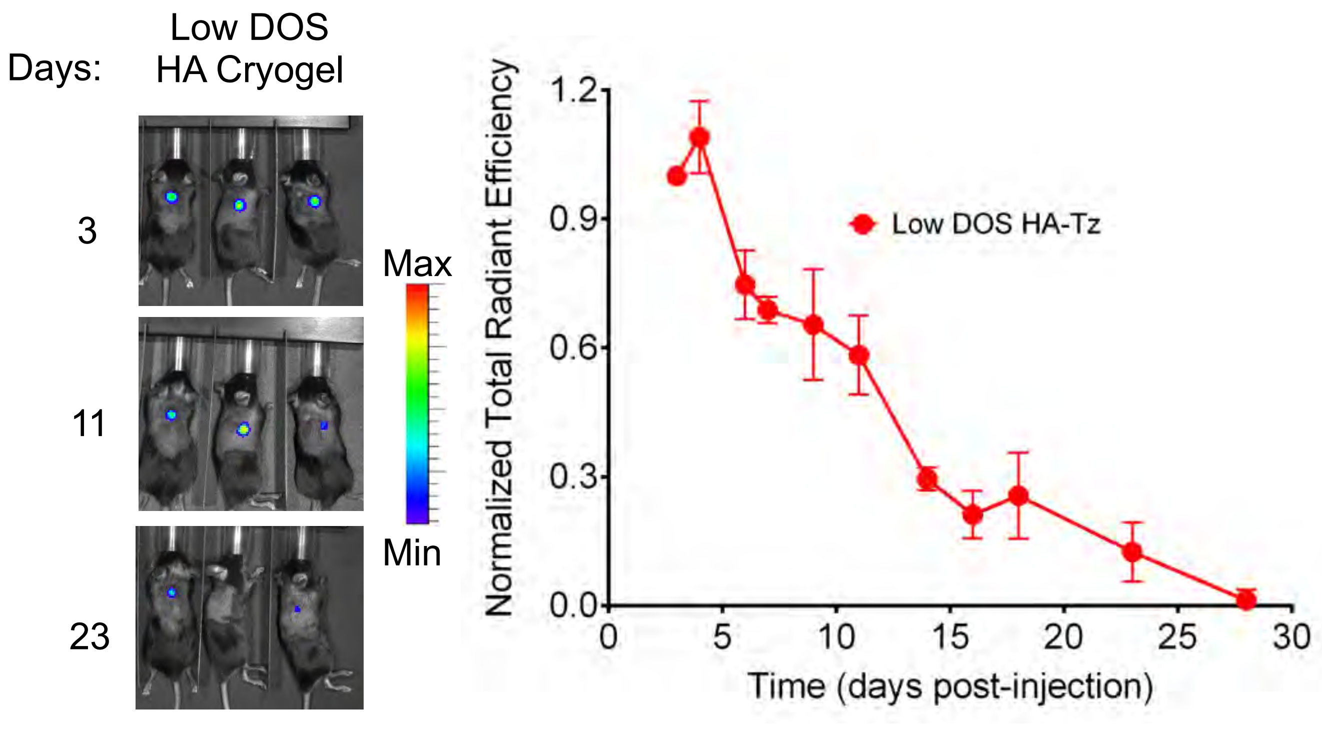

S2c

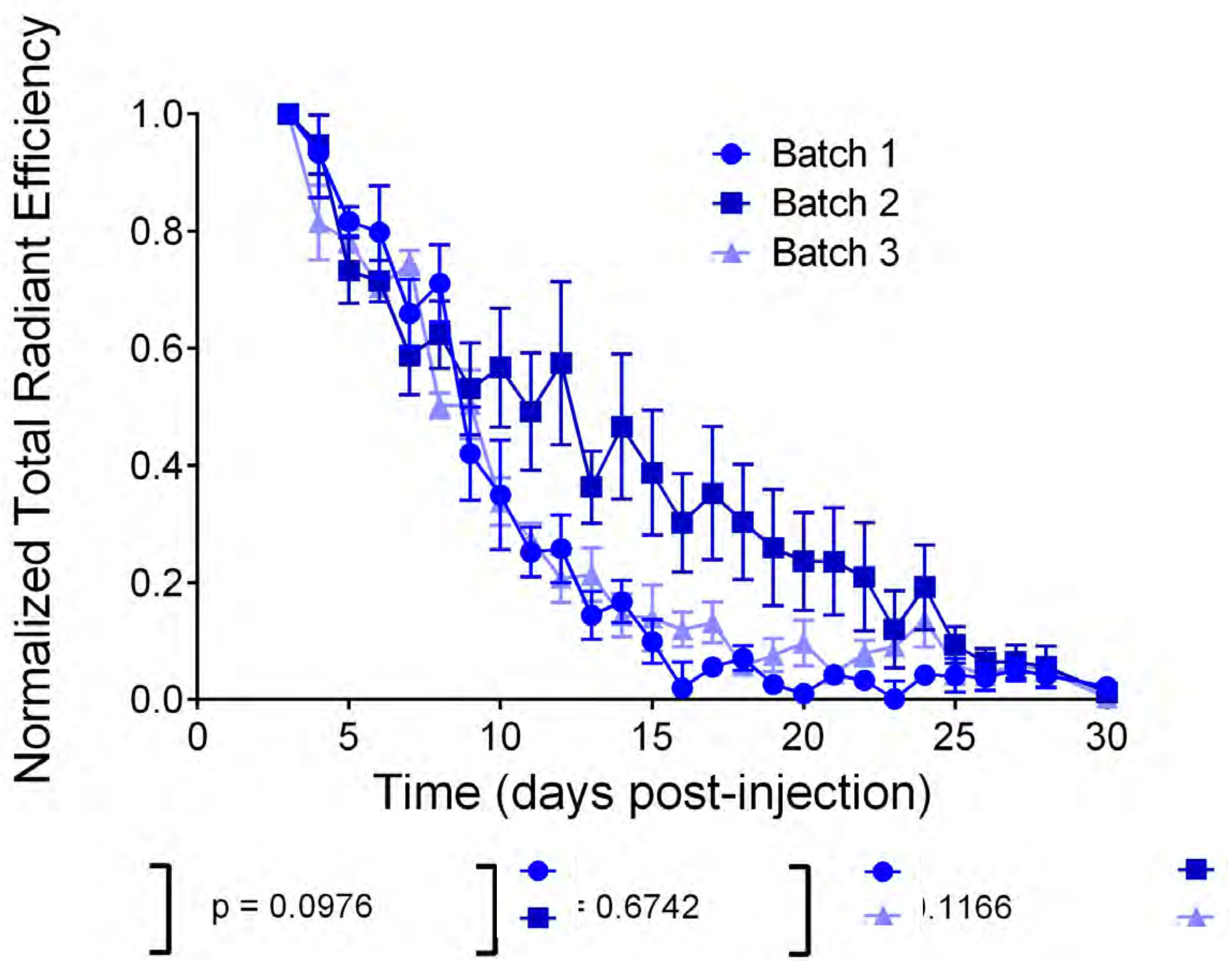

Supplementary Figure 3

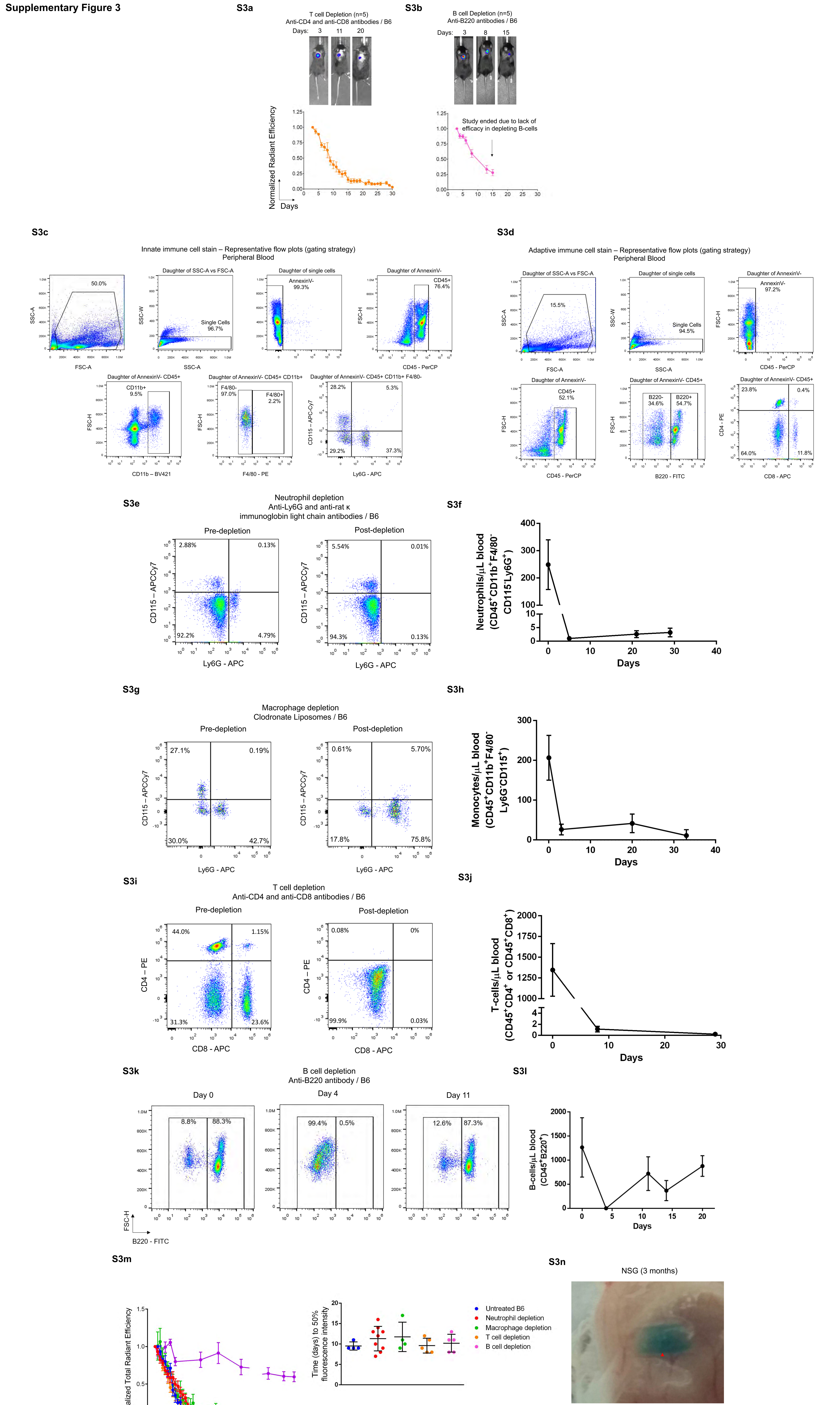

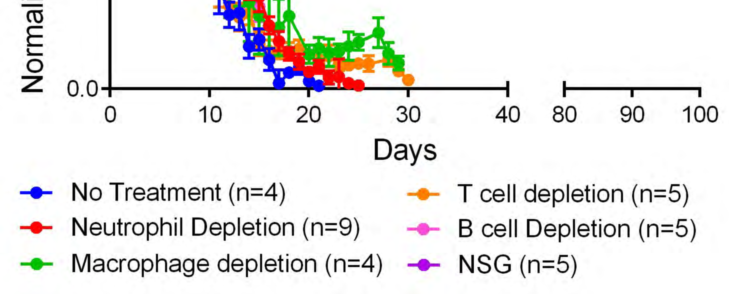

Comparison	F	Df (Degrees of freedom)	P value
Untreated B6 vs neutrophil depletion	9	$F_{8,3}$	0.1300
Untreated B6 vs macrophage depletion	12.92	$F_{3,3}$	0.3036
Untreated B6 vs T cell depletion	3.3	$F_{4,3}$	0.9197
Untreated B6 vs B cell depletion	4.7	$F_{4,3}$	0.5453

Supplementary Figure 4

S4a

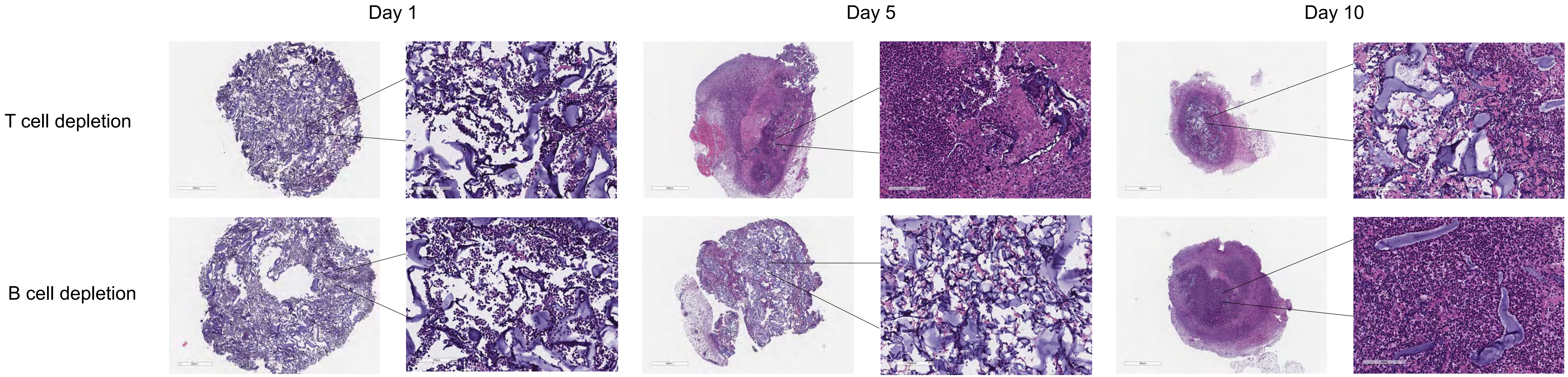

S4b

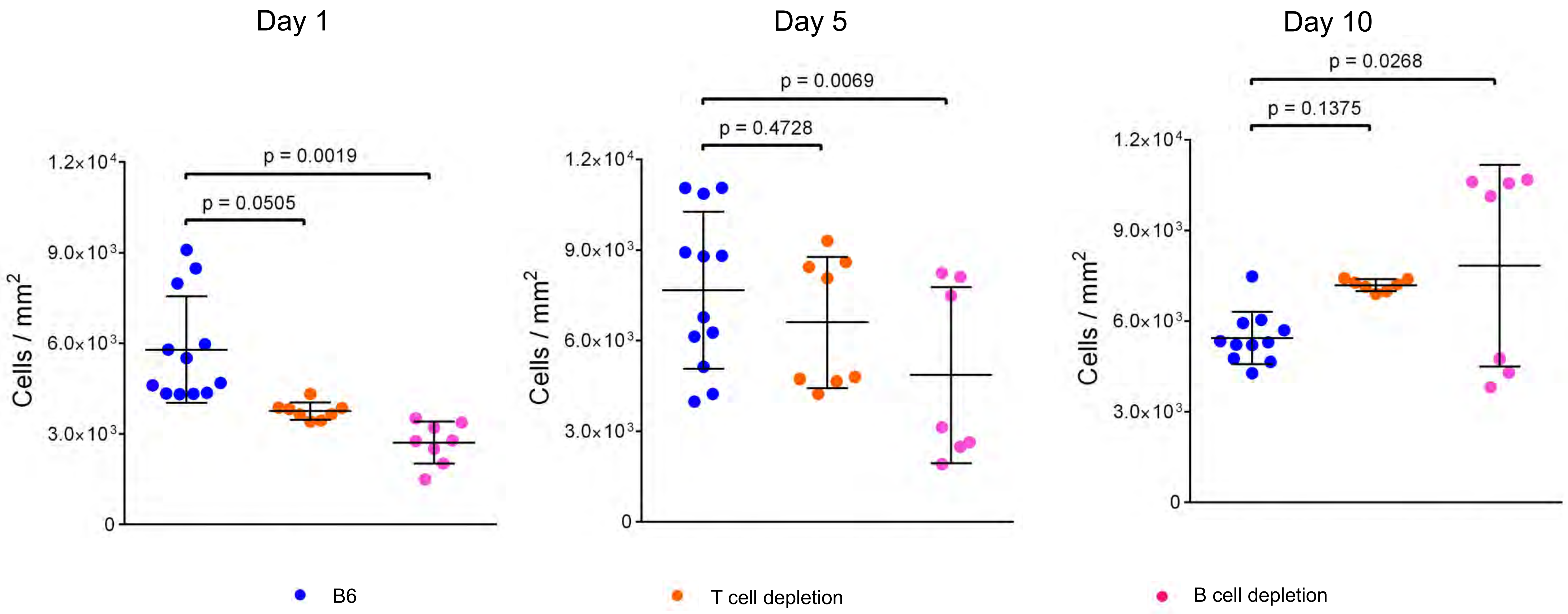

Supplementary Figure 5

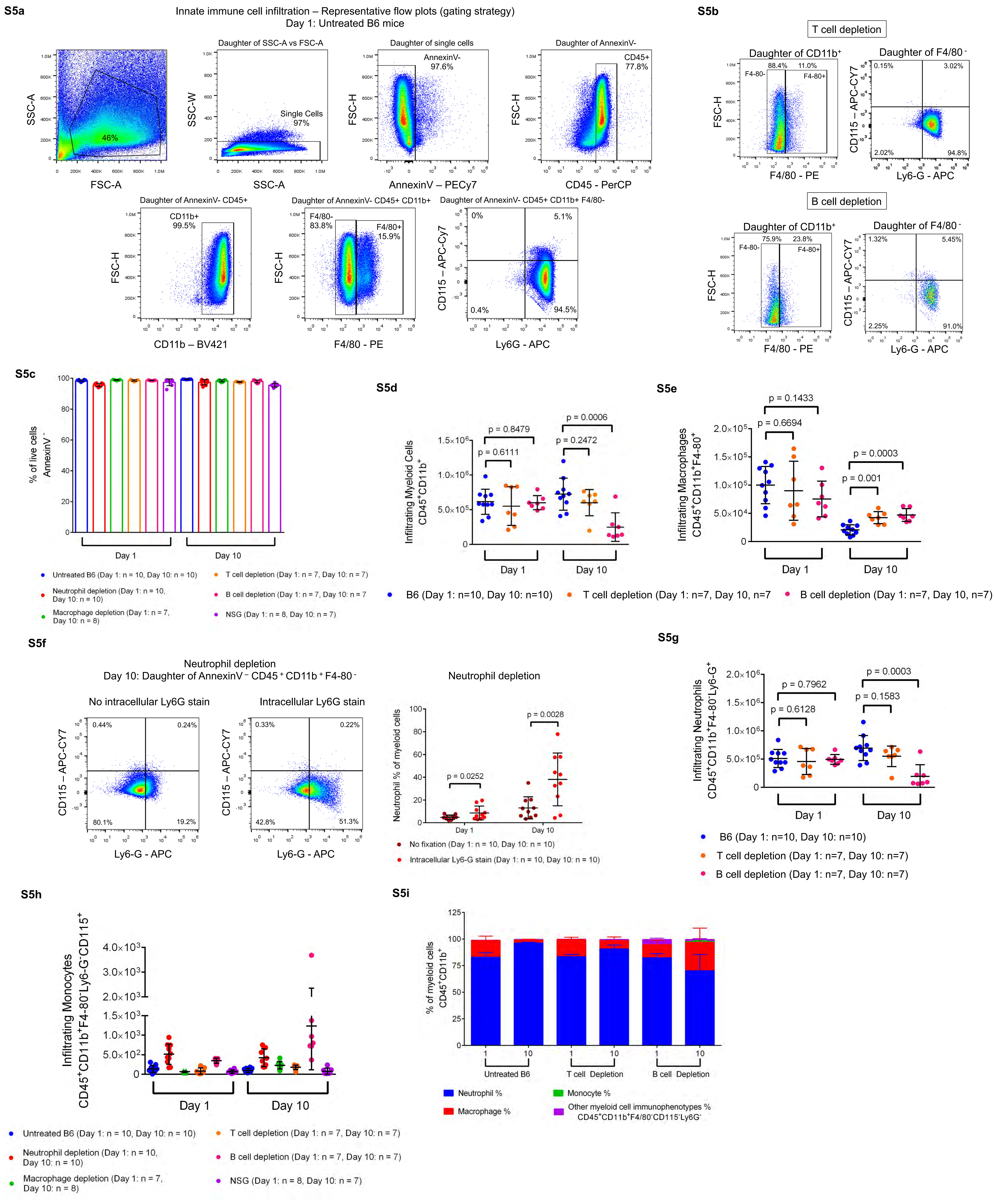

Supplementary Figure 6

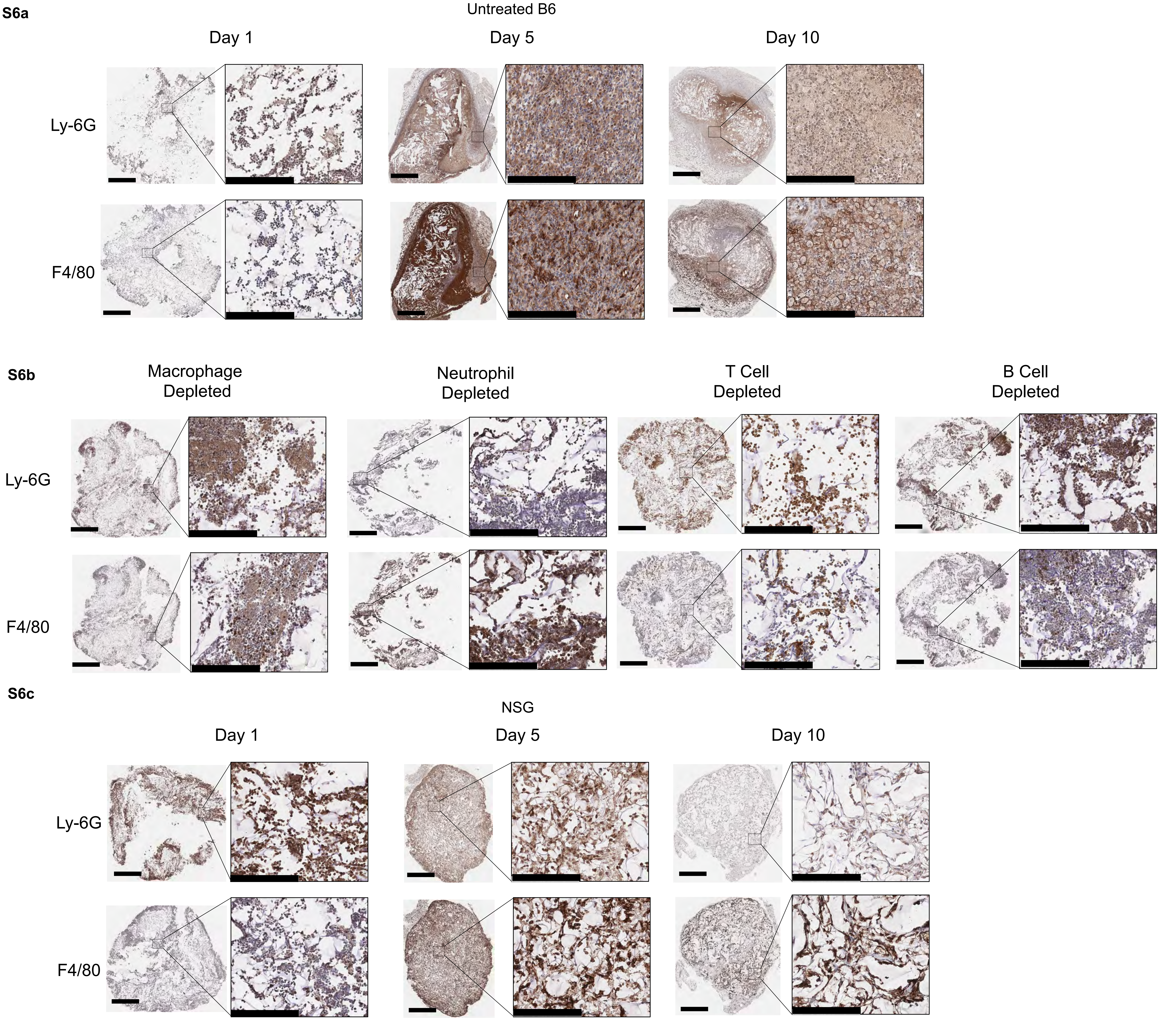

Supplementary Figure 7

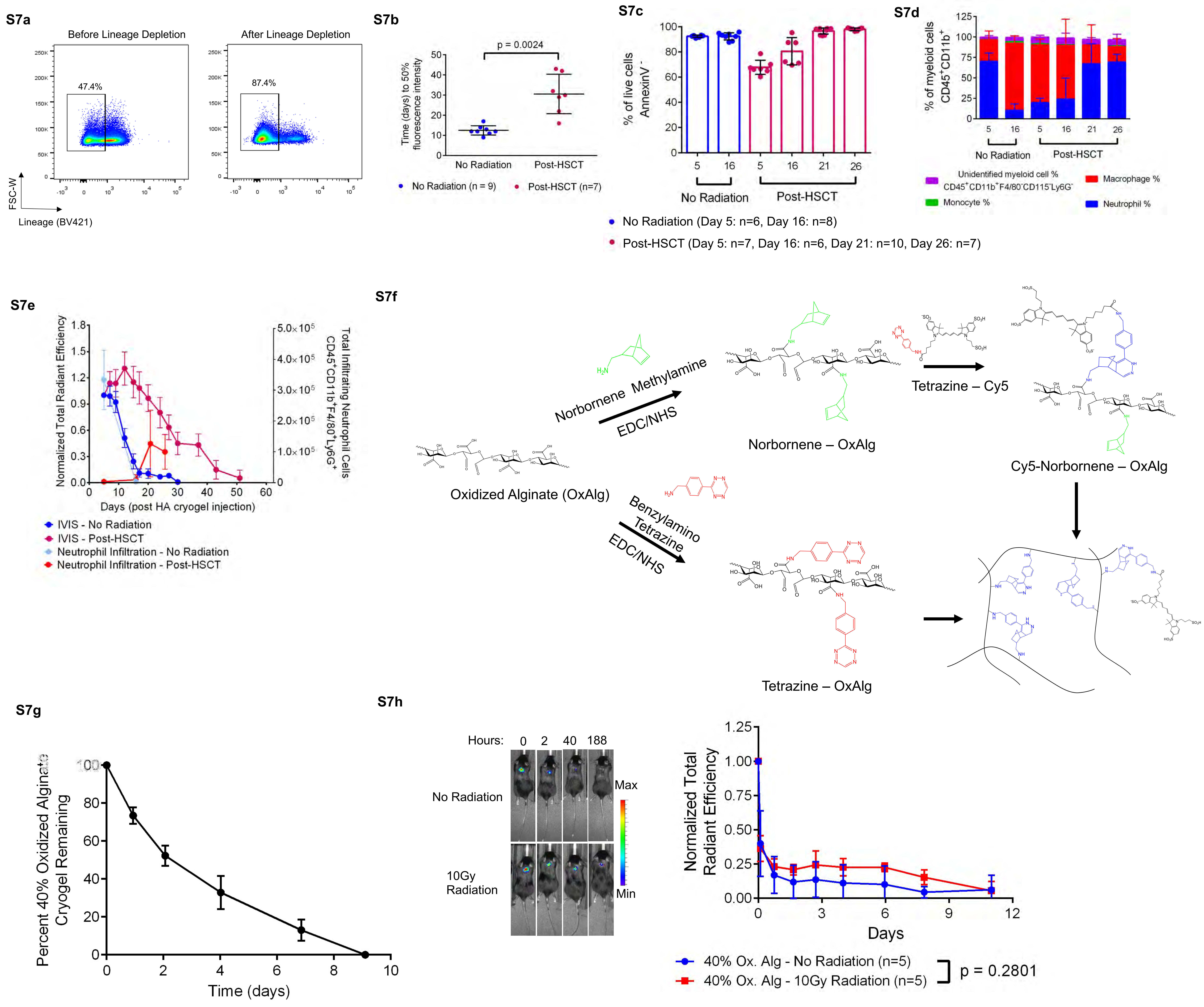

Supplementary Figure 8

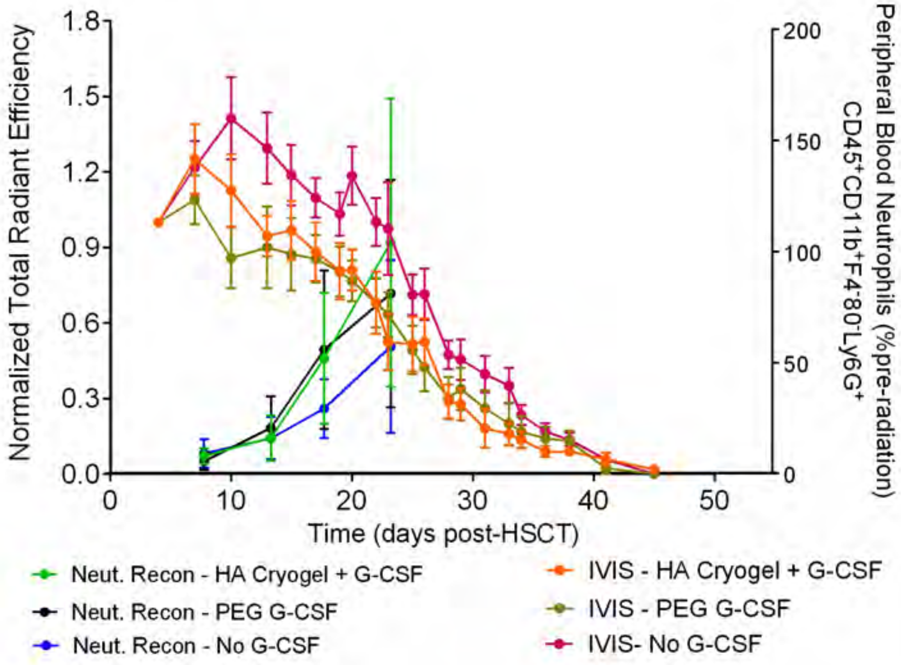

Supplement: Supplementary file 1 — Figure S1 Extended materials characterization of HA cryogels Figure S2 Extended characterization of Cy5‐HA cryogel degradation Figure S3 Extended characterization of Cy5‐HA cryogel degradation in immunodeficient mice Figure S4 Extended histomorphometric analysis of Cy5‐HA cryogels retrieved from T and B cell depleted mice Figure S5 Extended analysis of myeloid cell infiltration of Cy5‐HA cryogels retrieved from immunodeficient mice Figure S6 Extended immunohistochemical staining of Cy5‐HA cryogels retrieved from untreated B6 and NSG mice Figure S7 Extended quantification of post‐HSCT HA cryogel degradation Figure S8 Extended characterization of peripheral blood neutrophil reconstitution Table S1 Table S2 [file BTM2-8-e10309-s002.pdf]
