# Supplementary material for: Immune‐responsive biodegradable scaffolds for enhancing neutrophil regeneration
Source: Bioeng Transl Med. 2022 Apr 19;8(1):e10309. doi: 10.1002/btm2.10309 (PMC9842036; doi:10.1002/btm2.10309)
Supplement: Supplementary file 2 — Appendix S1: Supplementary figure captions and notes [file BTM2-8-e10309-s001.pdf]

Immune-responsive biodegradable scaffolds for enhancing neutrophil regeneration

Matthew D. Kerr[1, 2], David A. McBride[1, 2], Wade T. Johnson[1], Arun K. Chumber[1, 2], Alexander J. Najibi[4, 5], Bo Ri Seo[4, 5], Alexander G. Stafford[5], David T. Scadden[6,7,8], David J. Mooney[4, 5*] & Nisarg J. Shah[1,2,9*]

[1]Department of Nanoengineering, University of California, San Diego, La Jolla, CA 92093, USA

[2]Chemical Engineering Program, University of California, San Diego, La Jolla, CA 92093, USA

[4]John A. Paulson School of Engineering and Applied Sciences, Harvard University, Cambridge MA 02138

[5]Wyss Institute for Biologically Inspired Engineering, Harvard University, Cambridge, Massachusetts 02138

[6]Department of Stem Cell and Regenerative Biology, Harvard University, Cambridge, MA 02138, USA

[7]Harvard Stem Cell Institute, Cambridge, MA 02138, USA

[8]Center for Regenerative Medicine, Massachusetts General Hospital, Boston, MA 02114, USA

[9]Program in Immunology, University of California, San Diego, La Jolla, CA 92093, USA

Corresponding authors: Nisarg J. Shah and David J. Mooney

Supplementary Figure Captions

Fig. S1| Extended materials characterization of HA cryogels

(**a**) Volumetric swelling ratios for low- and high-DOS Cy5-HA cryogels. (**b**) Aqueous weight percentage of low- and high-DOS Cy5-HA cryogels. (**c**) Representative SEM image depicting low-DOS Cy5-HA cryogels. Top scale bar = 500μm, bottom scale bar = 100μm. (**d**) Average pore diameters of HA cryogels made from low- and high-DOS Cy5-HA cryogels measured from SEM images (20 measurements/cryogel, n = 3 each for low- and high-DOS HA cryogels). (**e**) Confocal microscopy images, overhead and side views, depicting low-DOS Cy5-HA cryogels both pre-injection and post-injection incubated with 10μm FITC-labeled microparticles. Scale bar = 100μm. (**f**) Quantification of confocal images showing penetration of 10μm FITC-labeled microparticles into both low- and high-DOS Cy5-HA cryogels pre- and post-injection. (**g**) Representative confocal microscopy images of low- and high-DOS Cy5-HA cryogels after thawing, after lyophilization, and after lyophilization and rehydration. (**h**) Average surface pore diameter of Cy5-HA cryogels measured from confocal images. Data in **a** and **b** represents mean ± s.d. of n=10 Cy5-HA cryogels. Data in **d** represents mean ± s.d. of n=3 Cy5-HA cryogels and was compared using student's t-test. Data in **f** represents mean ± s.d. of n=5 Cy5-HA cryogels. Data in **h** represents mean ± s.d. of n=3 HA cryogels and was compared using student's t-test.

Fig. S2| Extended characterization of Cy5-HA cryogel degradation

(**a**) Measuring low-DOS Cy5-HA cryogel degradation in vitro in hyaluronidase-2 (HYAL2) solution by quantification of Cy5-signal in supernatant at pre-determined timepoints normalized to total Cy5-signal in supernatant across all timepoints. (**b**) Representative IVIS fluorescence images of gel degradation in mice and measuring low-DOS Cy5-HA cryogel degradation in vivo by quantification of total radiant efficiency normalized to the initial day 3 timepoint. (**c**) Measuring Cy5-HA cryogel degradation by quantification of total radiant efficiency normalized to initial day 3 timepoint. Data in **a** represents mean ± s.d. of n=4 HA cryogels. Data in **b** represents mean ± s.e.m. of n=4 HA cryogels. Data in **c** represents mean ± s.d. of n=4-5 HA cryogels and were compared using two-way ANOVA with Bonferroni's multiple comparison test.

Fig. S3| Extended characterization of Cy5-HA cryogel degradation in immunodeficient mice

Representative IVIS fluorescence images of gel degradation and quantification by measuring total radiant efficiency normalized to initial day 3 timepoint of (**a**) T cell depleted B6 mice and (**b**) B cell depleted B6 mice. **c-d** Representative gating strategy to determine identity of (**c**) innate immune cells and (**d**) adaptive immune cells in peripheral blood. (**e**) Representative flow cytometry plot of peripheral blood neutrophils pre- and post-administration of neutrophil depleted mice and (**f**) peripheral blood neutrophil concentration. (**g**) Representative flow cytometry plot of peripheral blood monocytes pre- and post- administration clodronate liposomes to mice and (**h**) peripheral blood monocyte concentration. (**i**) Representative flow cytometry plot of peripheral blood T cells blood pre- and post- administration of anti-CD4 and anti-CD8 antibody treatment to mice and (**j**) peripheral blood T cell concentration. (**k**) Representative flow cytometry plot of peripheral blood B cells blood pre- and post- administration of anti-B220 antibody treatment to B6 mice and (**l**) peripheral blood B cell concentration. (**m**) Overlay of normalized total radiant efficiency curves and time to 50% fluorescence intensity of untreated B6, neutrophil depleted, macrophage depleted, T cell depleted, and B cell depleted mice. (**n**) Photograph of a Cy5-HA cryogel retrieved from NSG mice 3 months post-injection. Data in **a**, **b** represents mean ± s.e.m. of n=5 and are representative of at least two separate experiments. Data in **f**, **h**, **j**, **l** represents mean ± s.d. of n=4-5. Data in **m** represents mean ± s.d. of n=4-9 and were compared using student's t-test.

Fig. S4| Extended histomorphometric analysis of Cy5-HA cryogels retrieved from T and B cell depleted mice

(**a**) Hematoxylin and eosin (H&E) stain of explanted Cy5-HA cryogels from T cell depleted and B cell depleted mice at days 1, 5, and 10. Scale bar left = 800μm, scale bar right = 100μm. (**b**) Analysis of H&E stains to quantify cellular density in Cy5-HA cryogel. Data in **b** represents mean ± s.d. of n = 7-12 histological sections and was compared using student's t-test.

Fig. S5| Extended analysis of myeloid cell infiltration of Cy5-HA cryogels retrieved from immunodeficient mice

(**a**) Representative gating strategy to determine identity of innate immune cell infiltrates of HA cryogel. (**b**) Representative flow cytometry plots gated to determine cellular identity of $CD45^+$ $CD11b^+$ $F4/80^+$ (macrophage) cells, $CD45^+$ $CD11b^+$ $F4/80^-$ $Ly6G^+$ (neutrophil) cells, and $CD45^+$ $CD11b^+$ $F4/80^-$ $Ly6G^-$ $CD115^+$ (monocyte) cells T cell depleted and B cell depleted mice. (**c**) Percent of $AnnexinV^-$ (live) cells within Cy5-HA cryogels one and ten days after implant from flow cytometry analysis. **d-e** Quantification of total number of (**d**) myeloid cells and (**e**) macrophages infiltrating Cy5-HA cryogels in untreated B6 mice, T cell depleted mice, and B cell depleted mice. (**f**) Representative flow cytometry plots from neutrophil depleted mice with and without intracellular Ly6G staining. Plotted data assessing neutrophils as a percentage of total myeloid cells ($CD45^+CD11b^+$) with and without intracellular Ly6G staining. (**g**) Quantification of total number of neutrophils infiltrating Cy5-HA cryogels in untreated B6 mice, T cell depleted mice, and B cell depleted mice. **h,i** Quantification of total number of (**h**) monocytes and (**i**) infiltrating immune cell lineages plotted as a percentage of myeloid cells in untreated, T cell depleted, and B cell depleted mice. Data in **e** represents mean ± s.d. of n = 10. Data in **c, d, e, g, h, i** represents mean ± s.d. of n = 7-10 and are representative of at least two separate experiments. Data in **d, e, f, g** were compared using student's t-test.

Fig. S6| Extended immunohistochemical staining of Cy5-HA cryogels retrieved from untreated B6 and NSG mice

(**a**) Immunohistochemistry (IHC) staining for Ly6G (neutrophils, top, scale bar = 1mm) and F4/80 (macrophages, bottom, scale bar = 60μm) of Cy5-HA cryogels excised from untreated B6 mice 1-, 5-, and 10-days after injection. IHC was conducted on the same Cy5-HA cryogels as in Fig. 2e. (**b**) IHC staining for Ly6G (top, scale bar = 1mm) and F4/80 (bottom, scale bar = 60μm) of Cy5-HA cryogels excised from macrophage depleted, neutrophil depleted, T cell depleted, and B cell depleted B6 mice 1-day after injection. IHC was conducted on the same Cy5-HA cryogels as in Fig. 2e. (**c**) IHC staining for Ly6G (top, scale bar = 1mm) and F4/80 (bottom, scale bar = 60μm) of Cy5-HA cryogels

excised from NSG mice 1-, 5-, and 10-days after injection. IHC was conducted on the same Cy5-HA cryogels as in Fig. 2e.

Fig. S7| Extended quantification of post-HSCT HA cryogel degradation

(**a**) Representative flow cytometry plots of bone marrow before and after lineage depletion. (**b**) Time to 50% fluorescence intensity of Cy5-HA cryogels in non-irradiated and post-HSCT mice. (**c**) Percent of AnnexinV- (live) cells within Cy5-HA cryogels 5- and 16-days post injection in non-irradiated mice and 5-, 16-, 21-, and 26-days post-injection in post-HSCT mice. (**d**) Infiltrating immune cell lineages plotted as a percentage of myeloid cells in non-irradiated and post-HSCT mice. (**e**) Overlay of Cy5-HA cryogel degradation and neutrophil infiltration into in non-irradiated and post-HSCT mice from Fig. 4b, f. (**f**) Schematic for tetrazine (Tz) and norbornene (Nb) functionalization of oxidized alginate (OxAlg), Cy5 functionalization of Nb functionalized OxAlg, and crosslinking of Tz functionalized HA with Cy5 functionalized OxAlg. (**g**) Measuring Cy5-OxAlg cryogel degradation in vitro by quantifying the Cy5-signal in supernatant at pre-determined timepoints normalized to total Cy5-signal in supernatant across all timepoints. (**h**) Representative in vivo imaging system (IVIS) fluorescence images of gel degradation in mice and measuring Cy5-tagged 40% oxidized alginate cryogel degradation in vivo by quantification of total radiant efficiency normalized to initial 2-hour timepoint. IVIS Images are on the same scale and analyzed using Living Image Software. Data in **b** represents n=7-9 Cy5-HA cryogels, is representative of at least two separate experiments and were compared using student's t-test. Data in **c, d** represents mean ± s.d. of n=6-10 Cy5-HA cryogels and is representative of at least two separate experiments. Data in **g** represents mean ± s.d. of n=5 Cy5-OxAlg cryogels. Data in **h** represents mean ± s.e.m. of n=5 Cy5-OxAlg cryogels and were compared using two-way ANOVA with Bonferroni's multiple comparison test.

Fig. S8| Extended characterization of peripheral blood neutrophil reconstitution

Overlay of peripheral blood neutrophil recovery and Cy5-HA cryogel degradation in post-HSCT mice from Fig. 5 e, f.

Supplementary Note 1

The second order rate constant (k) for Tz-Nb reaction has been previously estimated to be 1.3 - 1.7 $M^{-1}s^{-1}$ at 21°C (room temperature).[1,2] In our system, for high-DOS Tz-HA and Nb-HA the concentration is 0.55mM at the start of the reaction. The reaction rate can be calculated as:

$$rate = k[Nb][Tz] \quad (1)$$

We calculated the rate of reaction to be about 40μM/s from equation 1 and the time to completion to be about 46.3 minutes at 21°C. In our system, the initial temperature is 4°C and therefore the actual time for completion of the reaction would be significantly longer.

As the solution cools and freezes during the crosslinking process, we estimated the freezing time. First, we determined the energy required to freeze 30uL of HA solution starting from 4°C and ending at 0°C using:

$$Q = \frac{\Delta H_i - \Delta H_f}{\rho V} \quad (2)$$

The energy required to freeze 30μL HA solution from 4°C to 0°C is 10.5J[3]. Since the HA solution is very dilute (0.6 wt%), we have approximated the enthalpy of formation and density to that of water.

To calculate the freezing time, we need to estimate the rate of energy extraction from the HA solution. Since the teflon cryomold is pre-cooled to -20°C and rests on a metal shelf in the freezer, we can estimate the rate of freezing using the thermal conductivity of teflon, thickness of teflon (25mm), and conductive heat transfer area (5.75mm) using equation 3. To simplify the analysis, we assume that convective heat loss at air-cryogel interface is negligible.

$$rate\ of\ heat\ transfer\ = \frac{kA\Delta T}{L} \qquad (3)$$

The rate of heat transfer is calculated to be 0.010J/s and therefore the time to reach 0°C is ~16.8 minutes. Since we are ignoring conductive heat loss through the edge of the cryogels and convective heat loss through the top, the calculated time represents an overestimation for the freezing time but is still significantly below that of the time to reaction completion. We have also experimentally verified that a 30uL drop of Tz-HA/Nb-HA solution freezes in about 10 minutes.

References:

1. Vrabel M, Kölle P, Brunner KM, Gattner MJ, López-Carrillo V, de Vivie-Riedle R, Carell T. Norbornenes in inverse electron-demand Diels-Alder reactions. Chemistry. 2013;19(40):13309-12.
2. Devaraj, N. K., Weissleder, R., & Hilderbrand, S. A. (2008). Tetrazine-based cycloadditions: application to pretargeted live cell imaging. Bioconjugate chemistry, 19(12), 2297–2299. https://doi.org/10.1021/bc8004446
3. https://www.engineeringtoolbox.com/saturated-ice-steam-d_970.html

Supplementary Note 2

To model G-CSF pharmacokinetics (PK) in the blood, the release profile of G-CSF from HA cryogels in post-HSCT mice was modeled as a piecewise function (**Fig. SN2-1**). Phase 1 was used to estimate G-CSF release from days 0-12 and phase 2 was used for days 12-40. We adopted parameters from previous reports[1,2].

$$Phase\ 1: G_{Release} = 0.7848 + 0.2112\ exp(-0.006138\ t)$$

$$Phase\ 2: G_{Release} = 0.09537 + 0.6989\ exp(-0.004115\ [t - 286])$$

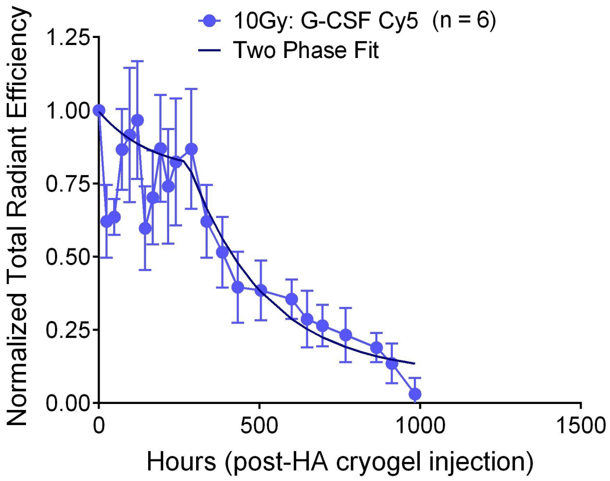

Figure SN2-1. Two-phase curve fit of G-CSF release from HA cryogel post-HSCT (as depicted in **Fig. 5b**).

The model takes into account endogenous production of G-CSF and assumes two HA cryogels loaded with 1μg of G-CSF each as sources. Renal clearance and internalization by neutrophil progenitors are consumption terms (**Equations 1,2** and **Fig. SN2-2a**).

$$Equation\ 1: \frac{dG_{SubQ}}{dt} = \frac{dG_{Release}}{dt}\frac{1}{V_s} - k_a G_{SubQ}$$

$$Equation\ 2: \frac{dG_{Blood}}{dt} = zk_a G_{SubQ} + G_{Prod} - \underbrace{k_{ren} G_{Blood}}_{G_{Renal}} - \underbrace{NXk_{int} \frac{G_{Blood^2}}{G_{Blood^2} + k_D}}_{G_{Internal}}$$

Parameter	k_a	k_{ren}	k_{int}	G_{prod}	z	V_S	N	X	k_D
Value	0.56 h^{-1}	0.43 h^{-1}	4.8 h^{-1}	0.01 ng/mL/hr	30/2000	0.1 mL	Function of Time via reconstitution data	0.000246	1.44 ng/mL

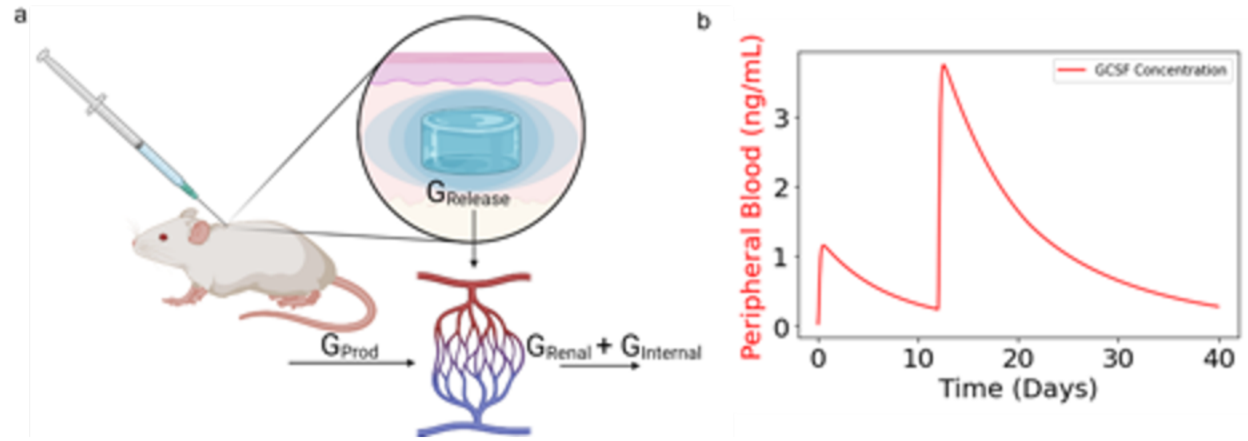

Figure SN2-2. (**a**) Schematic depicting sources of G-CSF production and clearance in peripheral blood. (**b**) Predicted G-CSF concentration in peripheral blood from two subcutaneously administered G-CSF encapsulated HA cryogels based on experimentally determined G-CSF release from HA cryogels.

The resulting G-CSF concentration in the peripheral blood of mice is estimated in **Fig. SN2-2b**. The code underlying the model can be found here:

References:

1. Craig M, Humphries AR, Nekka F, Bélair J, Li J, Mackey MC. Neutrophil dynamics during concurrent chemotherapy and G-CSF administration: Mathematical modelling guides dose optimisation to minimise neutropenia. Journal of Theoretical Biology. 2015; 385: 77-89.
2. Krzyzanski W, Wiczling P, Lowe P, Pigeolet E, Fink M, et al. Population Modeling of Filgrastim PK-PD in Healthy Adults Following Intravenous and Subcutaneous Administrations. The Journal of Clinical Pharmacology. 2010;50(59): 101S-112S.
